# Chameleonic Cages: Encapsulation of Anionic, Neutral, and Cationic Guest Species within [Fe_4_L_4_]^8+^ Tetrahedral Cages Synthesised from the *tris*(4‐aminophenyl)phosphate pro‐Ligand

**DOI:** 10.1002/chem.202402547

**Published:** 2024-10-29

**Authors:** Jas S. Ward, Paul E. Kruger

**Affiliations:** ^1^ MacDiarmid Institute for Advanced Materials and Nanotechnology School of Physical and Chemical Sciences University of Canterbury 8041 Christchurch New Zealand; ^2^ Current address: University of Jyvaskyla Department of Chemistry 40014 Jyväskylä Finland

**Keywords:** Host-guest systems, Phosphates, Self-assembly, Supramolecular chemistry, Transition metals

## Abstract

An adaptable Fe(II) tetrahedral cage, [Fe_4_L_4_][BF_4_]_8_ (L=*tris*(4‐(((E)‐pyridin‐2‐ylmethylene)amino)phenyl) phosphate), has been synthesised *via* self‐assembly. By modulating the orientation of its pendant P=O groups, the cage was found to be capable of encapsulating anionic, neutral, and cationic guests, which were confirmed in the solid state via single‐crystal X‐ray diffraction (SCXRD) and in solution by high‐resolution mass spectroscopy (HR‐MS), as well as by NMR (^1^H, ^19^F, ^31^P) studies where possible.

## Graphical abstract

An adaptable Fe(II) tetrahedral cage, [Fe_4_L_4_][BF_4_]_8_, based on a *tri*‐substituted phosphate ligand has been synthesised *via* self‐assembly. By modulating the orientation of its pendant P=O groups, the cage is capable of encapsulating anionic, neutral, and cationic guests, which was confirmed in the solid state via SCXRD.

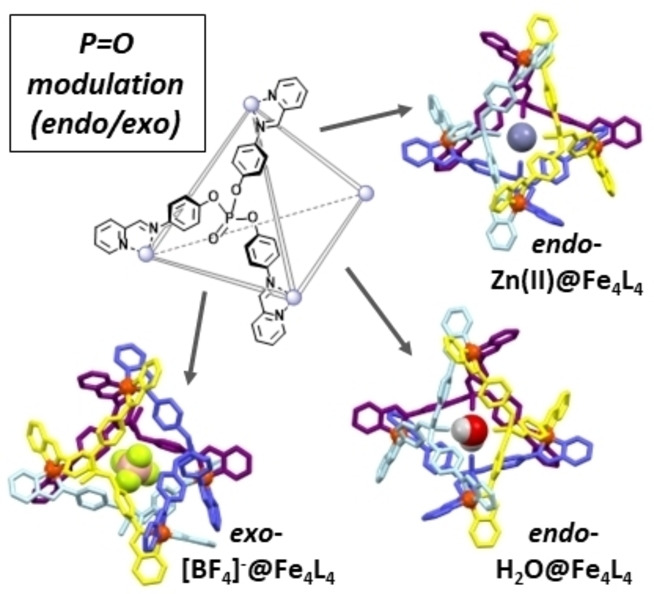

## Introduction

The synthesis of supramolecular cages through self‐assembly has produced a myriad of species with various topologies, many of which are capable of accommodating guest molecules within their internal voids,[^1^, ^2^, ^3^, ^4^, ^5^] with tetrahedral cages being one of the most studied.[^6^, ^7^] Traditionally, selectivity has been based around the interplay between the host and guest to achieve the best synergy of size, shape, bonding, and electronic factors, with the effects being largely intuitive. Nevertheless, the ability to create dynamic supramolecular architectures, with the ability to adapt to different conditions and substrates, is an intriguing and worthwhile prospect for future host‐guest system design. Toward this endeavour, it is the functionalisation of the internal micro‐environment of such complexes which might offer new possibilities for novel host‐guest behaviour.[^8^, ^9^]

The incorporation of main‐group functionalities into supramolecular cages, with their varied and specific non‐bonding interactions (*e. g*., hydrogen bonding, chalcogen bonding, and dipole‐dipole interactions), are of particular interest given their potential to facilitate stronger and more selective host‐guest chemistry.[^10^, ^11^] There have been few reports of the incorporation of phosphorus functionalities into supramolecular scaffolds to date,[^12^, ^13^, ^14^, ^15^] but inspired by the partitioning of the void space within cages using the pendant P=O substituent of P(O)(OR)_3_ based species in porous organic cage (POC) systems,^[16]^ a new class of adaptable cages was envisioned. However, unlike the reported POC system which possessed a structurally constrained *endo*/*endo* configuration for the two P=O substituents, the phosphate core would be designed to be flexible and therefore capable of being responsive to external conditions. The adaptable nature of the pendant P=O groups dynamically switching between *endo* or *exo* configurations would potentially facilitate control of the pore and/or void size, and therefore as a direct consequence the size and surface polarity of the cavity would be altered, affecting the selectivity of the guests encapsulated.

Based on a prior report of a porous organic cage synthesised from *tris*(4‐formylphenyl)phosphate,^[16]^ a similar system from *tris*(4‐aminophenyl)phosphate was envisioned, which could then be complexed *via* metal‐directed self‐assembly through imine‐condensation. A variation of this, which featured [M_4_L_4_][BF_4_]_8_ (M=Fe^II^/Co^II^) cages incorporating pendant P=S and P=Se groups, has been previously described.^[15]^ The solution studies of those cages understandably revealed complicated mixtures due to the possible isomers with respect to the metal centres (*i. e*., homochiral [ΔΔΔΔ/ΛΛΛΛ], heterochiral [ΔΔΔΛ/ΔΛΛΛ], and achiral [ΔΔΛΛ]), in addition to the possible *endo*/*exo* configurations of the pendant P=A (A = S, Se) groups, for which five unique configurations exist. Single crystal X‐ray diffraction (SCXRD) studies conducted on these complexes did observe two different arrangements of the pendant groups, with an exclusively endohedrally‐orientated structure for the P=Se cage, and an *endo*/*endo*/*endo*/*exo* motif for the P=S cage. Whilst the sole *exo* P=S group of the corresponding cage was found to be notably lengthened in the solid state due to coordination, unfortunately limitations in the data prevented identification of the coordinated species.

## Results and Discussion

**Figure 1:**
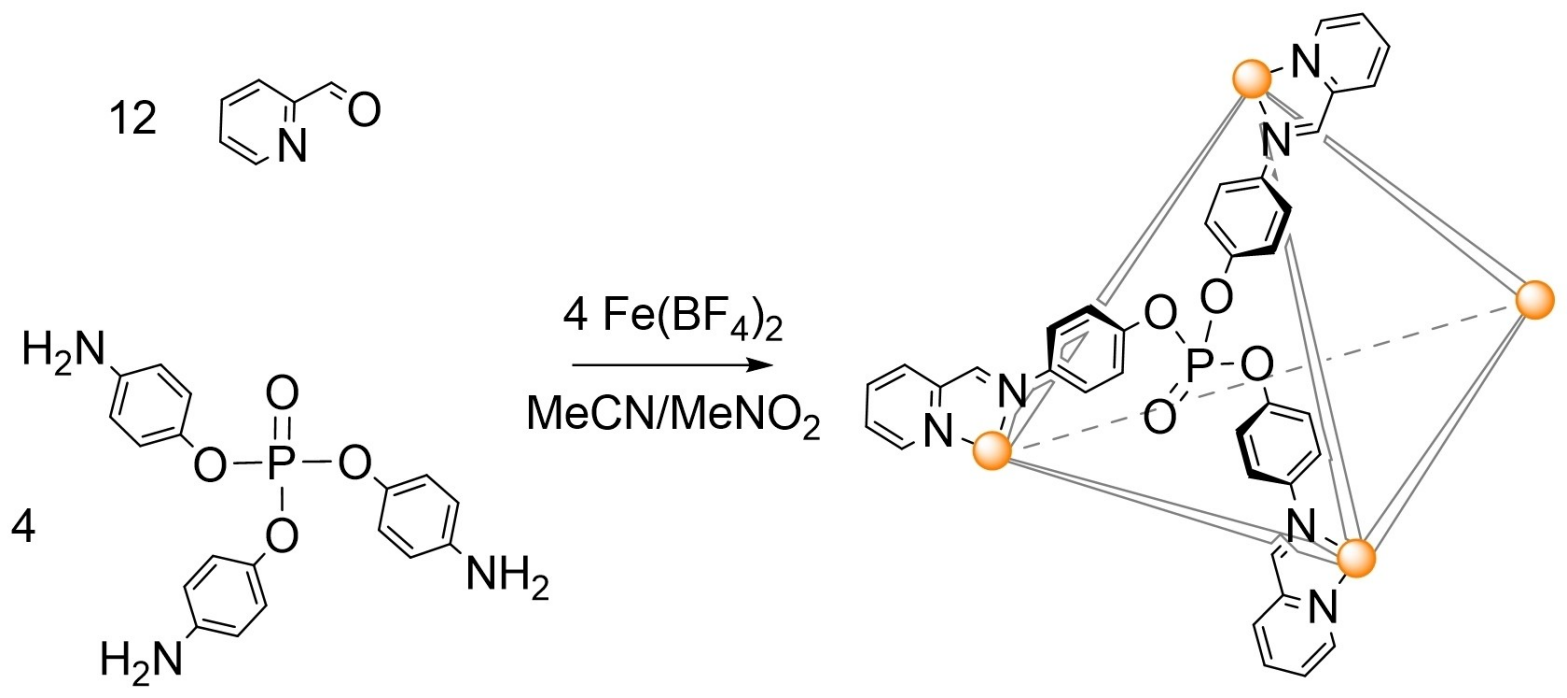
The sub‐component self‐assembly of *tris*(4‐aminophenyl)phosphate (**PL1**), 2‐pyridinecarboxaldehyde, and Fe(BF_4_)_2_ in acetonitrile or nitromethane to yield the corresponding facially‐capped [Fe_4_L_4_]^8+^ tetrahedron.

**Figure 2:**
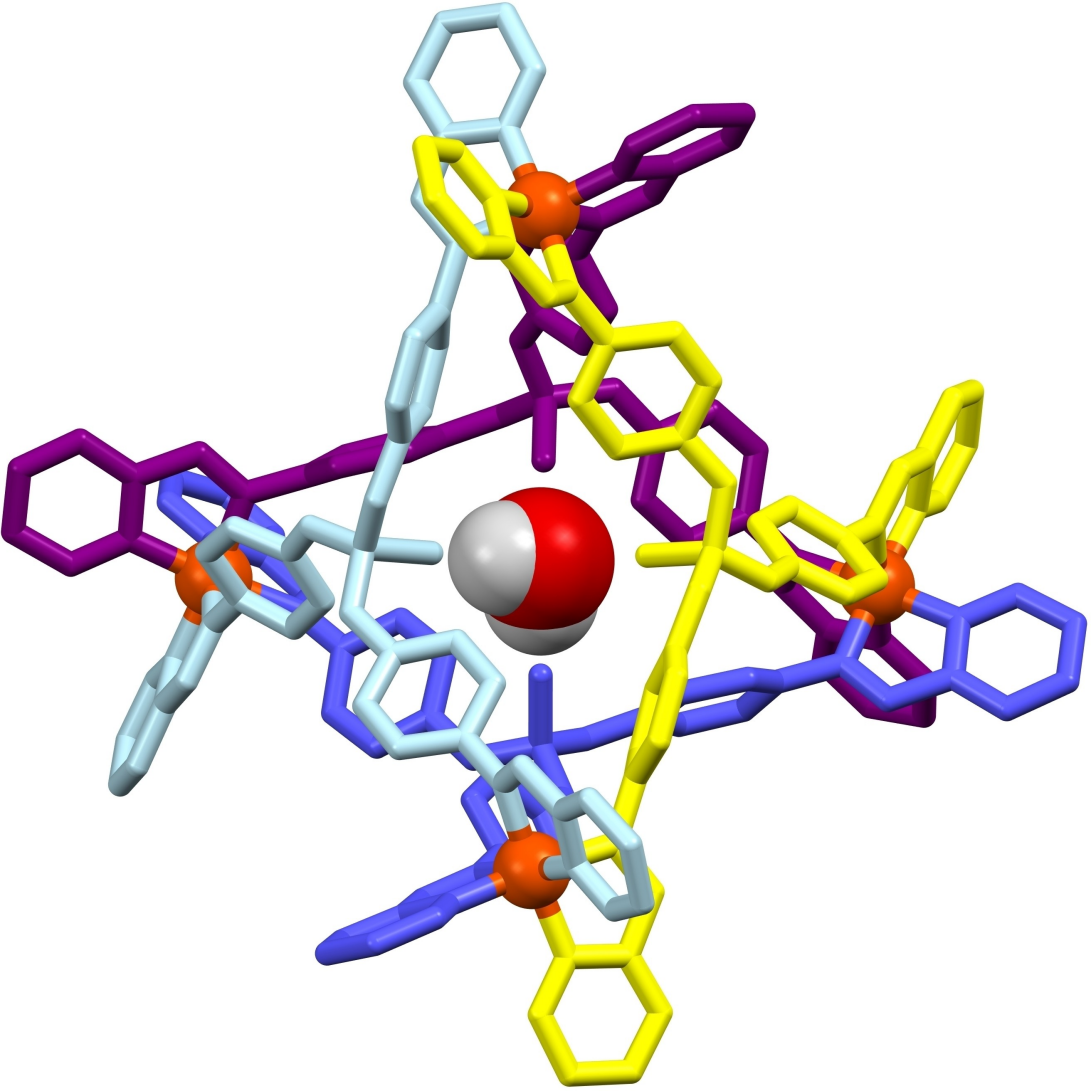
The molecular structure of **H_2_O@[Fe_4_L_4_][BF_4_]_8_** (one position of the symmetry‐disordered H_2_O molecule is shown; cage hydrogen atoms and anions omitted for clarity; the four ligands are block coloured for clarity).

In contrast to the P=S and P=Se tri‐amine pro‐ligands, which necessitated a protecting group strategy to be utilised in their synthesis,^[15]^ a more straightforward strategy starting from *tris*(4‐nitrophenyl)phosphate was followed,^[17]^ which was then converted to the desired pro‐ligand *tris*(4‐aminophenyl)phosphate (**PL1**) *via* a simple hydrogenation using H_2_ and a Pd/C catalyst in dry methanol. The metal‐directed self‐assembly reaction of **PL1**, 2‐pyridinecarboxaldehyde and Fe(BF_4_)_2_ ⋅ 6(H_2_O), in nitromethane or acetonitrile in a 4 : 12 : 4 stoichiometry, was found to generate the desired [Fe_4_L_4_][BF_4_]_8_ tetrahedral cage (Figure 1; L=*tris*(4‐(((E)‐pyridin‐2‐ylmethylene)amino)phenyl) phosphate) as a deep purple coloured species, characteristic of low spin Fe(II), as expected for the strong‐field nature of the pyridyl‐imine coordination sphere.^[18]^ Crystallisation of the filtered crude reaction solution yielded dark purple cube crystals, which were confirmed by single crystal X‐ray crystallography to be **H_2_O@[Fe_4_L_4_][BF_4_]_8_** (Figure 2).

**Figure 3:**
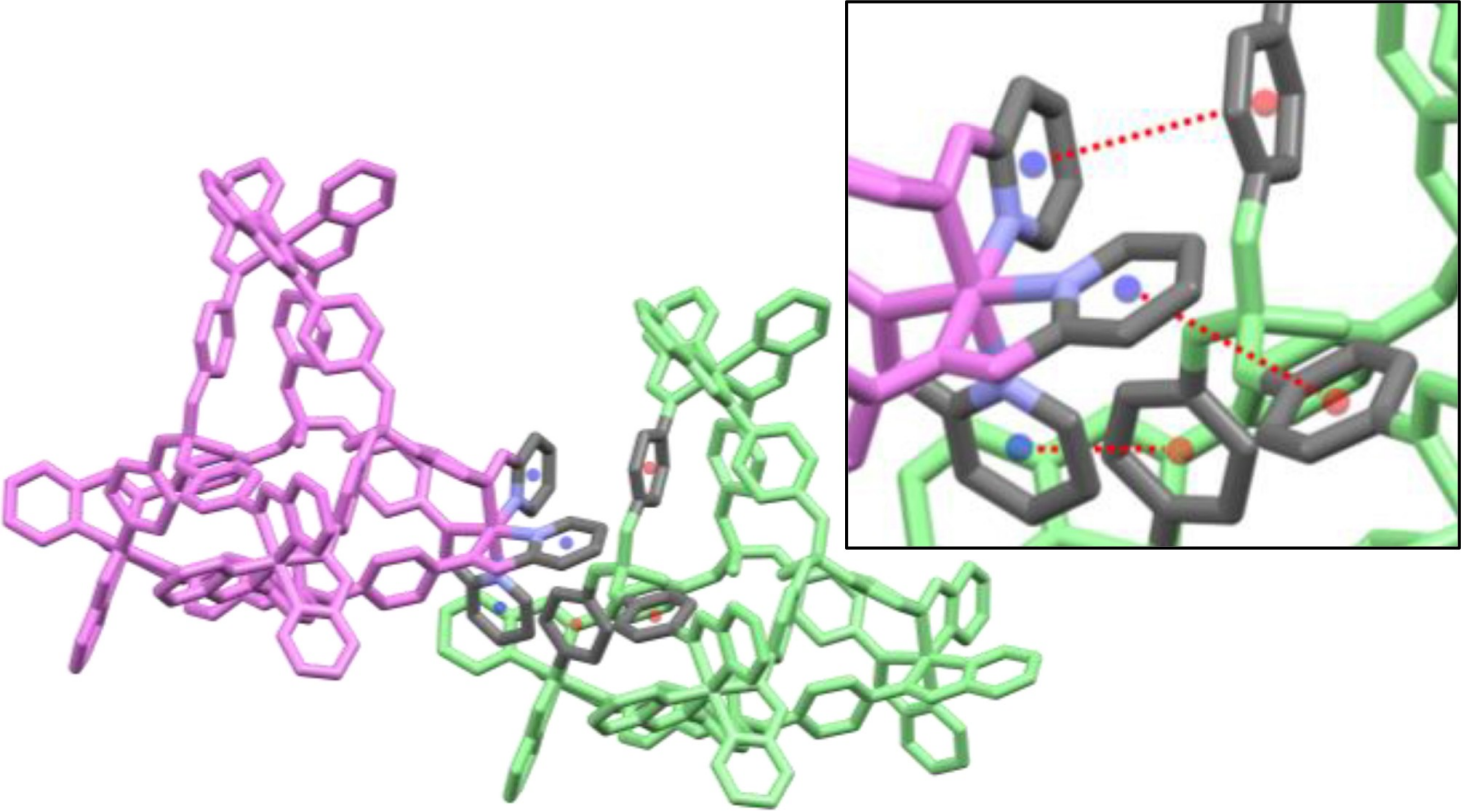
The efficient cubic packing of two [Fe_4_L_4_]^8+^ moieties of **H_2_O@[Fe_4_L_4_][BF_4_]_8_** via π‐stacking of three crystallographically identical phosphate phenylene substituents and three pyridyl head groups (and expansion as inset; Key: grey=carbon; blue=nitrogen). All centroid(phenylene; red)⋅⋅⋅centroid(pyridyl; blue) distances are equivalent due to the cubic symmetry with distances of 4.32 Å.

The molecular structure of **H_2_O@[Fe_4_L_4_][BF_4_]_8_** was found to be in the non‐centrosymmetric *I*23 cubic space group, a rare highly‐symmetric space group with only a few other tetrahedral cages being reported to date.[^8^, ^19^, ^20^, ^21^, ^22^, ^23^, ^24^, ^25^] The non‐centrosymmetric space group meant that only the homochiral ΛΛΛΛ diastereoisomer was present in the solved crystal, though presumably the ΔΔΔΔ diastereoisomer was also present in equal ratio but not observed crystallographically. The cubic symmetry of the complex is a result of the efficient (and notably solvate‐free) packing that is present in the structure, where each tetrahedral cage is directly interacting with eight other adjacent cages. This packing arrangement is made possible by each octahedral Fe(II) centre (of each corner of the tetrahedron) being closely nestled into the facial phosphate node of a neighbouring cage, encouraged by every phenylene substituent demonstrating π‐bonding to a pyridyl ring of an adjacent cage, with centroid‐to‐centroid distances of 4.32 Å (Figure 3).

The encapsulated water molecule possesses symmetry‐dictated disorder over 6 positions such that it is always hydrogen bonding to two of the four pendant P=O groups within the cage. The formally P=O group bond length of 1.68(5) Å is longer than the expected range, and is significantly longer than the formally single bond of the three P−OR substituents (*cf*. 1.47(2) Å), where this elongation is likely the result of hydrogen bonding between the host and guest inside the cage, further complicated by the enforced high symmetry of the cubic space group.

Dissolution of the isolated crystals for characterisation by NMR spectroscopy (^1^H, ^19^F, ^31^P) repeatedly gave highly complex spectra that could not be deciphered, and inconsistent with the solid‐state symmetry determined crystallographically, indicating the cubic symmetry was not retained in solution. However, solution studies did confirm the presence of the tetrahedral cage *via* high resolution mass spectroscopy, with the observation of isotopic manifolds consistent for [Fe_4_L_4_]^8+^ species (Figures S5–S9). However, no peaks consistent with retention of the encapsulated water could be identified.

**Figure 4:**
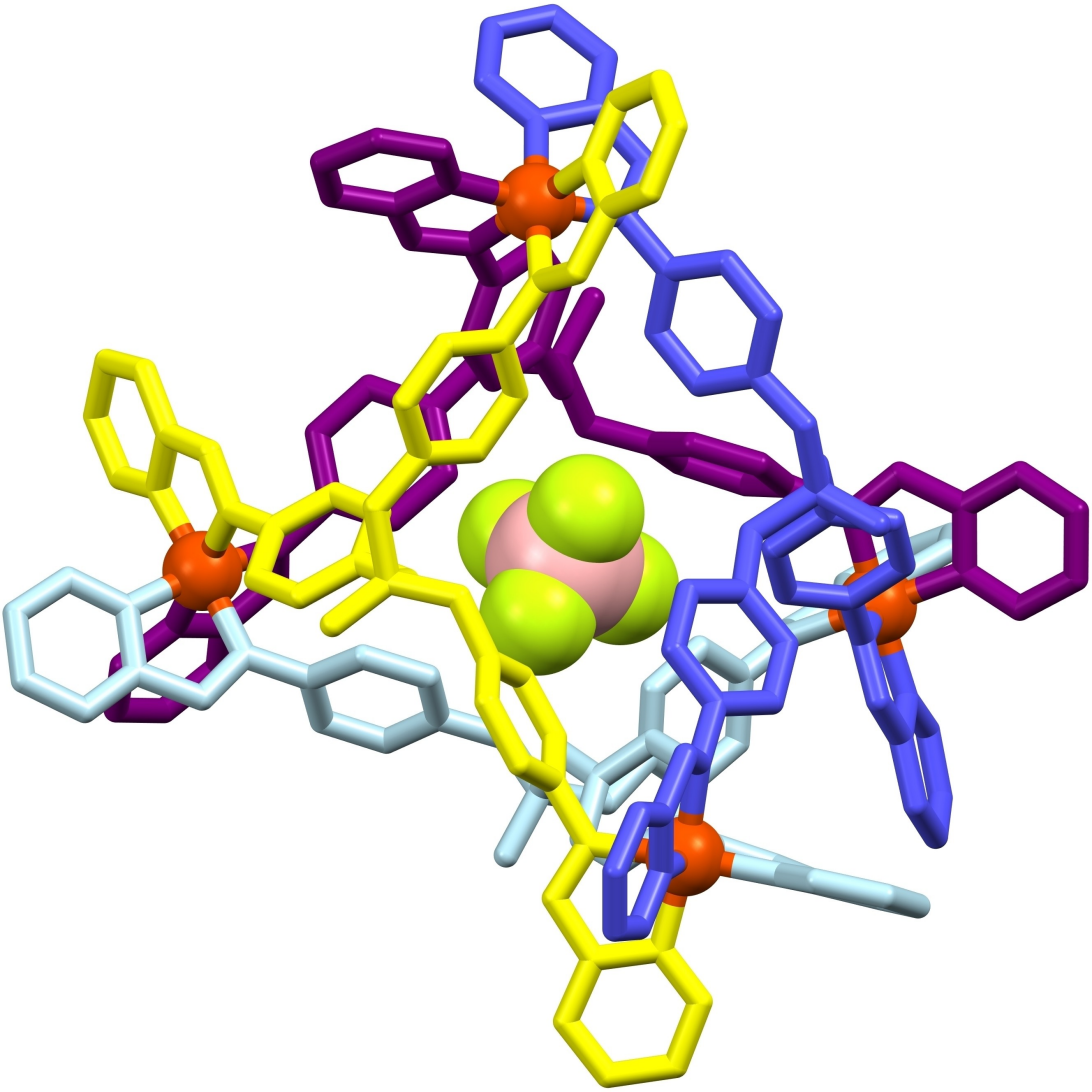
The molecular structure of **[BF_4_]^−^@[Fe_4_L_4_][BF_4_]_7_** (hydrogen atoms, solvates and non‐encapsulated anions omitted for clarity; the four ligands are block coloured for clarity).

Based on the initial success of isolating **H_2_O@[Fe_4_L_4_][BF_4_]_8_**, doping studies were conducted involving a range of additives, with the intention of replacing the encapsulated water, or even of altering the tetrahedral cage topology (viz. *endo*/*exo*) with respect to the pendant P=O groups. Doping of the filtered crude solution with a stoichiometric amount of 0.25 molL^−1^ aqueous [NH_4_][BF_4_] solution was intended to provide a more symmetry‐accommodating hydrogen bond donor ([NH_4_]^+^) to replace the opportunistically encapsulated H_2_O. However, single crystal X‐ray diffraction confirmed the presence of an encapsulated [BF_4_]^−^ anion as **[BF_4_]^−^@[Fe_4_L_4_][BF_4_]_7_**, accommodated by the tetrahedral cage adopting an all *exo* configuration with respect to the four P=O substituents (Figure 4).

The encapsulation of an adventitious counterion is a commonly observed outcome for supramolecular cages,[^26^, ^27^, ^28^] though in this instance it is the cause of anion encapsulation that is of interest. As with the solid‐state structure of the H_2_O encapsulation, the non‐centrosymmetric space group meant that only the homochiral (ΔΔΔΔ) diastereoisomer was present. Whilst the role of the [NH_4_][BF_4_] is unclear, is it presumed that the [NH_4_]^+^ is necessary to create the exclusively *exo* configuration of the cage, due to a lack of any notable interactions of the [BF_4_]^−^ within the cavity, and the failure to prepare this configuration without [NH_4_][BF_4_] despite the abundance of [BF_4_]^−^ anions in the synthesis of **H_2_O@[Fe_4_L_4_][BF_4_]_8_**. The tetra‐*exo* configuration of **[BF_4_]^−^@[Fe_4_L_4_][BF_4_]_7_** prevented the highly efficient and solvate‐free packing previously observed for **H_2_O@[Fe_4_L_4_][BF_4_]_8_**. This observation is somewhat surprising given that the general motif of the Fe(II) centre nestling into the facial phosphate nodes in **H_2_O@[Fe_4_L_4_][BF_4_]_8_** (*vide supra*) is mirrored in the packing of **[BF_4_]^−^@[Fe_4_L_4_][BF_4_]_7_**, with P⋅⋅⋅Fe intermolecular distances of 5.280(2)‐5.489(2) Å in **[BF_4_]^−^@[Fe_4_L_4_][BF_4_]_7_** being either shorter or comparable to those found in **H_2_O@[Fe_4_L_4_][BF_4_]_8_** (*cf*. 5.451(9) Å). However, despite the proximity, there were no favourable intermolecular π‐interactions observed between the phosphate phenylene rings and the pyridyl head groups as previously observed for **H_2_O@[Fe_4_L_4_][BF_4_]_8_**, obstructed in **[BF_4_]^−^@[Fe_4_L_4_][BF_4_]_7_** by the four *exo*‐orientated P=O substituents.

The four crystallographically independent P=O groups were, as expected, shorter than their P‐OR counterparts with bond lengths ranging from 1.448(5)‐1.460(5) Å and 1.558(5)‐1.578(6) Å, respectively. These values indicate that the P=O bond lengths of 1.68(5) Å in **H_2_O@[Fe_4_L_4_][BF_4_]_8_** might not be a true representation of the strength of the bonding in that system, or that the exclusively *endo* configuration of **H_2_O@[Fe_4_L_4_][BF_4_]_8_**, coupled with the cubic‐enforced symmetry, is participating in strong hydrogen bonding.

**Figure 5:**
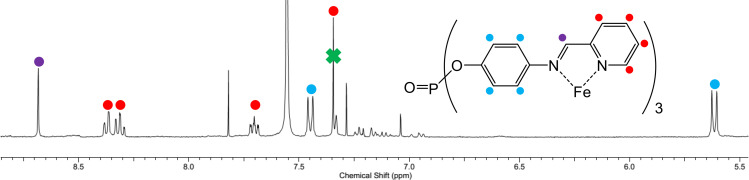
The aromatic region of the ^1^H NMR spectrum (in CD_3_CN) of the tetrahedral cage **[BF_4_]^−^@[Fe_4_L_4_][BF_4_]_7_** (annotated with the structure of the ligand; the benzene peak from crystallisation that overlaps with one of the pyridyl doublets has been labelled with a green cross). Colour key: purple=imine; blue=phenylene; red=pyridyl.

Solution studies performed on **[BF_4_]^−^@[Fe_4_L_4_][BF_4_]_7_** in deuterated acetonitrile found one dominant product in the ^1^H NMR resonances observed, which matched the symmetry of the complete phosphate ligand (Figure 5), which included approximately matching integrals (as a trace impurity was also observed). The identity of the complete phosphate ligand was confirmed by the absence of the pro‐ligand peaks (which were at significantly lower frequency), and secondly by the characteristic imine resonance observed as a singlet at 8.68 ppm. The ^19^F NMR spectrum also confirmed two different [BF_4_]^−^ anion environments at −151.6 and −149.8 ppm with integration being approximately 7 : 1, respectively. The ^31^P NMR spectrum found one resonance at −22.2 ppm, which was noticeably different than that observed for **PL1** (*cf*. δ_P_ −14.6 in CD_3_CN). Mass spectroscopy studies similarly confirmed the presence of the tetrahedral cage in solution, however, it was inconclusive as to whether any of these anions were encapsulated, or simply charge aggregated, by the cage.

The metal cation encapsulated species, **[Cu]^+^@[Fe_4_L_4_][BF_4_]_9_** and **[Zn]^2+^@[Fe_4_L_4_][BF_4_]_10_**, were similarly synthesised by doping crude reaction mixtures with stoichiometric solutions (1 : 1 cage:dopant) of [Cu(MeCN)_4_][BF_4_] or Zn(BF_4_)_2_, respectively. The solid‐state structure of **[Cu]^+^@[Fe_4_L_4_][BF_4_]_9_** (Figure S2) indicated that the Cu(I) cation had only partial occupancy, with the remaining electron density being attributed to a water molecule, with refined occupancies nearing 50 : 50 % (and then manually rounded for the purposes of computational modelling). However, microanalysis of the isolated crystalline material confirms the composition as 1 : 1 Cu(I):cage, suggesting the Cu(I) encapsulation (unlike the encapsulated H_2_O) is possibly fluxional. Conversely, the Zn(II) dication in the solid‐state structure of **[Zn]^2+^@[Fe_4_L_4_][BF_4_]_10_** (Figure S3) was found to be fully occupied in the cavity, as confirmed by free refinement of its occupancy during refinement of the model. Both metal‐encapsulated complexes were found to be isostructural to the H_2_O‐encapsulated complex in the *I*23 space group, and observed as the homochiral ΛΛΛΛ [Cu(I)] and ΔΔΔΔ [Zn(II)] diastereoisomers. Nevertheless, solution studies on these metal‐encapsulated species proved inconclusive, with complicated NMR spectra being repeatedly observed from isolated crystals redissolved in acetonitrile for both **[Cu]^+^@[Fe_4_L_4_][BF_4_]_9_** and **[Zn]^2+^@[Fe_4_L_4_][BF_4_]_10_**, with the added caveat that solutions of **[Cu]^+^@[Fe_4_L_4_][BF_4_]_9_** were no longer a deep purple, but a pinkish colour, upon dissolution, possibly indicating decomposition or another as‐yet unknown process in action. However, mass spectroscopic studies on redissolved crystals of **[Zn]^2+^@[Fe_4_L_4_][BF_4_]_10_** did confirm the presence of the tetrahedral cage in solution, though once again no fragments could be identified that demonstrated the encapsulated Zn(II) dication was retained.

## Conclusions

In conclusion, a main‐group tetrahedral cage has been synthesised from the *tris*(4‐aminophenyl)phosphate pro‐ligand, 2‐pyridinecarboxaldehyde, and Fe(BF_4_)_2_ ⋅ 6(H_2_O) *via* self‐assembly. The intrinsic *endo*/*exo* choice offered by the phosphate nodes with respect to their pendant P=O groups has enabled an adaptable supramolecular system capable of encapsulating anionic ([BF_4_]^−^), neutral (H_2_O), and cationic [Cu(I), Zn(II)] guest molecules. The identities of the tetrahedral cages were confirmed in the solid state by SCXRD, which also established the presence of the encapsulated guests, and in solution primarily by high resolution mass spectroscopy, but also by ^1^H, ^19^F, and ^31^P NMR studies for **[BF_4_]^−^@[Fe_4_L_4_][BF_4_]_7_**.

## Conflict of Interests

The authors declare no conflict of interest.

1

## Supporting information

As a service to our authors and readers, this journal provides supporting information supplied by the authors. Such materials are peer reviewed and may be re‐organized for online delivery, but are not copy‐edited or typeset. Technical support issues arising from supporting information (other than missing files) should be addressed to the authors.

Supporting Information

Supporting Information

Supporting Information

Supporting Information

Supporting Information

Supporting Information

## Data Availability

The data that support the findings of this study are available in the supplementary material of this article.
