# Supplementary material for: Chameleonic Cages: Encapsulation of Anionic, Neutral, and Cationic Guest Species within [Fe4L4]8+ Tetrahedral Cages Synthesised from the tris(4‐aminophenyl)phosphate pro‐Ligand
Source: Chemistry. 2024 Oct 29;30(67):e202402547. doi: 10.1002/chem.202402547 (PMC11609896; doi:10.1002/chem.202402547)
Supplement: Supplementary file 4 — Supporting Information [file CHEM-30-e202402547-s001.pdf]

Chemistry–A European Journal

Supporting Information

Chameleonic Cages: Encapsulation of Anionic, Neutral, and Cationic Guest Species within $[Fe_4L_4]^{8+}$ Tetrahedral Cages Synthesised from the *tris*(4-aminophenyl)phosphate pro-Ligand

Jas S. Ward* and Paul E. Kruger*

Chameleonic Cages: Encapsulation of Anionic, Neutral, and Cationic Guest Species within $[Fe_4L_4]^{8+}$ Tetrahedral Cages Synthesised from the *tris*(4-aminophenyl)phosphate pro-Ligand

Jas S. Ward*[a,b] and Paul E. Kruger*[a]

[a] Department of Chemistry & MacDiarmid Institute for Advanced Materials and Nanotechnology, University of Canterbury, Private Bag 4800, Christchurch 8041, New Zealand.
[b] Current address: University of Jyvaskyla, Department of Chemistry, 40014 Jyväskylä, Finland.

E-mail: james.s.ward@jyu.fi, paul.kruger@canterbury.ac.nz

Supporting Information

Table of Contents

Experimental Details S2
General Considerations S2
Single Crystal X-ray Diffraction (SCXRD) Studies S2
Synthetic Procedures S2
SCXRD Tables S5
High Resolution Mass Spectroscopy S6
NMR Spectroscopy S17
References S24

Experimental Details

General Considerations

All reagents were used as received without further purification from Sigma Aldrich, BDH and Acros Organics. ^{1}H, ^{19}F and ^{31}P NMR spectroscopy were conducted on an Agilent 400 NMR spectrometer operating at 400 MHz for ^{1}H, 376 MHz for ^{19}F and 162 MHz for ^{31}P. Chemical shifts are described in parts per million (ppm) on the δ scale. High resolution mass spectra were recorded with a Bruker maXis 3G UHR-TOF mass spectrometer at the University of Canterbury. Microanalysis was carried out at the Campbell Microanalytical Laboratory, University of Otago, New Zealand.

Single Crystal X-ray Diffraction (SCXRD) Studies

Crystallographic data for compounds **PL1**, **$[BF_4]^-@[Fe_4L_4][BF_4]_7$**, **$[Cu]^+@[Fe_4L_4][BF_4]_9$** and **$[Zn]^{2+}@[Fe_4L_4][BF_4]_{10}$** were collected on an Oxford-Agilent SuperNova instrument with focused micro-source Cu Kα (λ = 1.5418 Å) radiation and ATLAS CCD area detector, with data reduction performed using *CrysAlis PRO*. The data for **$H_2O@[Fe_4L_4][BF_4]_8$** was collected at the Australian Synchrotron MX1 Beamline with silicon double crystal-monochromatised Mo Kα (λ = 0.7108 Å) radiation and ADSC Quantum 210r detector, with data reduction performed using *BluIce* and *XDS*.[1,2] All structures were solved using direct methods with SHELXT and refined on F^2 using all data by full matrix least-squares procedures with SHELXL within OLEX2.[3–5] Non-hydrogen atoms were refined with anisotropic displacement parameters. Hydrogen atoms were included in calculated positions with isotropic displacement parameters 1.2 times the isotropic equivalent of their carrier atoms.

Synthetic Procedures

Synthesis of *tris*(4-nitrophenyl)phosphate: Compound was prepared as previously reported in good yield (96%).[6] ^{1}H NMR (400 MHz, $CDCl_3$): 7.45 (d, $^{3}J_{HH}$ = 8 Hz, 6H), 8.32 (d, $^{3}J_{HH}$ = 8 Hz, 6H);‡ ^{31}P NMR (162 MHz, $CDCl_3$): -20.0 (s).

[‡ *Literature ($CDCl_3$): ^{1}H NMR (400 MHz, $CDCl_3$): 7.44 (dd, J = 9.3 and 1.1 Hz, 6H), 8.31 (dd, J = 9.2 and 0.6 Hz, 6H).*[6]]

Synthesis of *tris*(4-aminophenyl)phosphate (PL1): *Tris*(4-nitrophenyl)phosphate (0.200 g, 0.43 mmol) was suspended in dry MeOH (16 mL), then Pd/C catalyst (0.02 g, 10% by weight of precursor) was added, and the system immediately purged with vacuum and flushed with H_2 gas, which was repeated three times to ensure removal of air. The grey suspension was stirred under H_2 gas for 21 hours to give a black suspension that was filtered through celite to collect the Pd/C catalyst. All volatiles were removed from the very pale-yellow filtrate under reduced pressure to leave a crystalline light orange crystalline solid. Yield = 0.144 g (0.39 mmol, 90%). ^{1}H NMR (400 MHz, CD_3OD): 6.69 (d, $^{3}J_{HH}$ = 12 Hz, 6H), 6.91 (d, $^{3}J_{HH}$ = 12 Hz, 6H), NH_2 groups not observed in CD_3OD due to H/D exchange; ^{31}P NMR (162 MHz, CD_3OD): -15.1 (s). ^{1}H NMR (400 MHz, CD_3CN): 4.12 (s.br, 6H), 6.61 (d, $^{3}J_{HH}$ = 8 Hz, 6H), 6.92 (d, $^{3}J_{HH}$ = 8 Hz, 6H); ^{31}P NMR (162 MHz, CD_3CN): -14.6 (s). Acc. Mass: Found: m/z = 372.1120. Calcd. for $C_{18}H_{19}N_3O_4P$ 372.1108 $[M + H]^+$. Anal. Found: C, 57.32; H, 5.04; N, 11.12%. Calcd. for $C_{18}H_{18}N_3O_4P{\cdot}0.3(H_2O)$: C, 57.39; H, 4.98%; N, 11.15%. Crystal data: $C_{18}H_{18}N_3O_4P$, M_r = 371.32, T = 120.0(1) K, triclinic, space group P-1 (No. 2), a = 9.307(1) Å, b = 9.8081(8) Å, c = 10.3920(7) Å, α = 67.018(7)°, β = 81.591(8)°, γ = 83.423(8)°, V = 862.21(14) Å^3, Z = 2, D_{calcd} = 1.430 Mg m^{-3}, μ(Cu Kα) = 1.68 mm^{-1}, yellow plate, 0.03 x 0.18 x 0.27 mm, 6019 measured reflections with θ_{max} = 76.5°, 3490 independent reflections, 2837 with $I_o > 2\sigma(I_o)$, R_{int} = 0.037, 3490 data, 258 parameters, 1 restraint, GooF = 1.05, 0.27 > Δρ > -0.40 eÅ^{-3}, $R[F^2 > 2\sigma(F^2)]$ = 0.050, $wR(F^2)$ = 0.139, CCDC 1530372.

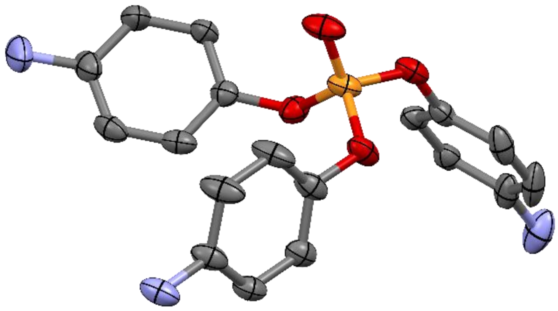

Figure S1. The molecular structure of *tris*(4-aminophenyl)phosphate (**PL1**; thermal ellipsoids at 50% probability, hydrogen atoms omitted for clarity).

General Procedure for $[Fe_4L_4][BF_4]_8$: *Tris*(4-aminophenyl)phosphate (14.9 mg, 0.04 mmol) and $Fe(BF_4)_2 \cdot 6(H_2O)$ (13.5 mg, 0.04 mmol) were suspended in acetonitrile (10 mL) or nitromethane (10 mL), followed by addition of 2-pyridinecarboxaldehyde (11.4 µL, 0.12 mmol) to immediately give a deep red coloured solution (that became a deep purple colour in <1 min). The reaction was stirred for 3 hours, filtered through a grade 3 or 4 sinter and the filtrate subsequently vapour diffused with an excess of appropriate anti-solvent to yield X-ray diffraction quality crystals after several days.
Stoichiometric amounts of dopants (1:1 dopant:cage, when applicable) were introduced as solutions of known concentrations after the filtration of the crude reaction mixture. Dopants: $[NH_4][BF_4]$ (0.25 $molL^{-1}$ solution in H_2O, 40 µL, 0.01 mmol), $[Cu(MeCN)_4][BF_4]$ (0.063 $molL^{-1}$ solution in acetone, 160 µL, 0.01 mmol), $Zn(BF_4)_2$ (0.25 $molL^{-1}$ solution in H_2O, 40 µL, 0.01 mmol).

H_2O@$[Fe_4L_4][BF_4]_8$: Crystals were grown by vapour diffusion of ethyl acetate (2 mL) into a nitromethane (1 mL) aliquot of the reaction mixture. Acc. Mass: Found: *m/z* = 1070.4965, Calcd. for $[Fe_4L_4 + 5(BF_4)]^{3+}$ 1070.4951; *m/z* = 781.1215, Calcd. for $[Fe_4L_4 + 4(BF_4)]^{4+}$ 781.1205; *m/z* = 607.4960, Calcd. for $[Fe_4L_4 + 3(BF_4)]^{5+}$ 607.4957; *m/z* = 409.0692, Calcd. for $[Fe_4L_4 + BF_4]^{7+}$ 409.0673. Found: C, 48.22; H, 3.54; N, 9.18%. Calcd. for $[C_{144}H_{108}Fe_4N_{24}O_{16}P_4][BF_4]_8 \cdot 6(H_2O)$: C, 48.31; H, 3.38; N, 9.39%. Crystal data: $C_{144}H_{108}Fe_4N_{24}O_{16}P_4 \cdot 8(BF_4) \cdot H_2O$, M_r = 3490.31, T = 100(2) K, cubic, space group *I*23, a = 21.010(2) Å, V = 9275(3) $Å^3$, Z = 2, D_{calcd} = 1.250 Mg m^{-3}, µ(Mo Kα) = 0.43 mm^{-1}, purple block, 0.10 x 0.10 x 0.10 mm, 33249 measured reflections with θ_{max} = 26.3°, 3141 independent reflections, 1816 with $I_o > 2\sigma(I_o)$, R_{int} = 0.130, 3141 data, 259 parameters, 220 restraints, GooF = 1.33, 0.46 > Δρ > -0.50 e$Å^{-3}$, $R[F^2 > 2\sigma(F^2)]$ = 0.118, $wR(F^2)$ = 0.367, CCDC 1530416.

$[BF_4]^-$@$[Fe_4L_4][BF_4]_7$: Crystals were grown by vapour diffusion of benzene (2 mL) or *m*-xylene (2 mL) into an acetonitrile (1 mL) aliquot of the reaction mixture. ^{1}H NMR (400 MHz, CD_3CN): 5.62 (d, $^3J_{HH}$ = 8 Hz, 24H), 7.34 (*presumed* doublet overlapped with C_6H_6, 12H), 7.45 (d, $^3J_{HH}$ = 8 Hz, 24H), 7.70 (t, $^3J_{HH}$ = 8 Hz, 12H), 8.31 (t, $^3J_{HH}$ = 8 Hz, 12H), 8.38 (d, $^3J_{HH}$ = 8 Hz, 12H), 8.68 (s, 12H). ^{19}F NMR (376 MHz, CD_3CN): -151.6 (s), -149.8 (s). ^{31}P NMR (162 MHz, CD_3CN): -22.2 (s). Acc. Mass: Found: *m/z* = 1070.4986, Calcd. for $[Fe_4L_4 + 5(BF_4)]^{3+}$ 1070.4951; *m/z* = 781.1235, Calcd. for $[Fe_4L_4 + 4(BF_4)]^{4+}$ 781.1205. Found: C, 46.04 H, 3.35; N, 8.81%. Calcd. for $[C_{144}H_{108}Fe_4N_{24}O_{16}P_4][BF_4]_8 \cdot 15(H_2O)$: C, 46.21; H, 3.72; N, 8.98%. Crystal data: $C_{144}H_{108}Fe_4N_{24}O_{16}P_4 \cdot 8(BF_4) \cdot 1.5(C_8H_{10}) \cdot 8(C_2H_3N)$, M_r = 3959.97, T = 120.0(1) K, orthorhombic, space group *Iba*2, a = 56.1140(8) Å, b = 28.1044(4) Å, c = 24.1794(3) Å, V = 38132.1(9) $Å^3$, Z = 8, D_{calcd} = 1.380 Mg m^{-3}, µ(Cu Kα) = 3.56 mm^{-1}, purple block, 0.11 x 0.32 x 0.57 mm, 181371 measured reflections with θ_{max} = 77.1°, 34718 independent reflections, 29090 with $I_o > 2\sigma(I_o)$, R_{int} = 0.104, 34718 data, 2491 parameters, 519 restraints, GooF = 1.02, 1.77 > Δρ > -0.90 e$Å^{-3}$, $R[F^2 > 2\sigma(F^2)]$ = 0.080, $wR(F^2)$ = 0.220, CCDC 1530371.

$[Cu]^+$@$[Fe_4L_4][BF_4]_9$: Crystals were grown by vapour diffusion of benzene (2 mL) into a nitromethane (1 mL) aliquot of the reaction mixture. Found: C, 45.57 H, 3.61; N, 8.60%. Calcd. for $[C_{144}H_{108}CuFe_4N_{24}O_{16}P_4][BF_4]_9 \cdot 10(H_2O)$: C, 44.48; H, 3.39; N, 8.84%. Crystal data: $C_{144}H_{108}Fe_4N_{24}O_{16}P_4 \cdot 8.5(BF_4) \cdot 0.5(Cu) \cdot 0.5(H_2O)$, M_r = 3556.48, T = 120.0(1) K, cubic, space group *I*23, a = 20.9262(10) Å, V = 9163.7(13) $Å^3$, Z = 2, D_{calcd} = 1.289 Mg m^{-3}, µ(Cu Kα) = 3.70 mm^{-1}, purple block, 0.09 x 0.17 x 0.23 mm, 12837 measured reflections with θ_{max} = 43.3°, 1105 independent reflections, 921 with $I_o > 2\sigma(I_o)$, R_{int} = 0.092, 1105 data, 194 parameters, 226 restraints, GooF = 2.19, 0.65 > Δρ > -0.59 e$Å^{-3}$, $R[F^2 > 2\sigma(F^2)]$ = 0.170, $wR(F^2)$ = 0.445, CCDC 1530417.

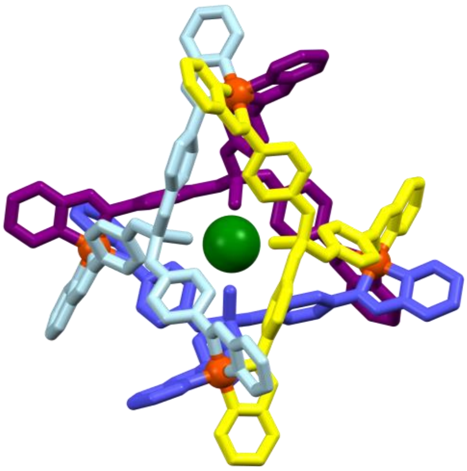

Figure S2. The molecular structure of **$[Cu]^+$@$[Fe_4L_4][BF_4]_9 \cdot H_2O$@$[Fe_4L_4][BF_4]_8$** (minor guest, anions, and hydrogen atoms omitted for clarity; thermal ellipsoids at 50% probability).

$[Zn]^{2+}$@$[Fe_4L_4][BF_4]_{10}$: Crystals were grown by vapour diffusion of chloroform (2 mL) into a nitromethane (1 mL) aliquot of the reaction mixture. Acc. Mass: Found: *m/z* = 409.0702, Calcd. for $[Fe_4L_4 + BF_4]^{7+}$ 409.0673. Found: C, 44.56 H, 3.35; N, 8.60%. Calcd. for $[C_{144}H_{108}Fe_4N_{24}O_{16}P_4Zn][BF_4]_{10}\cdot 10(H_2O)$: C, 44.51; H, 3.30; N, 8.65%. Crystal data: $C_{144}H_{108}Fe_4N_{24}O_{16}P_4\cdot 10(BF_4)\cdot Zn$, M_r = 3711.29, T = 120.0(1) K, cubic, space group *I*23, a = 21.0609(12) Å, V = 9341.8(16) $Å^3$, Z = 2, D_{calcd} = 1.319 Mg m^{-3}, μ(Cu Kα) = 3.77 mm^{-1}, purple block, 0.08 x 0.14 x 0.17 mm, 32886 measured reflections with θ_{max} = 75.3°, 3166 independent reflections, 1717 with $I_o > 2\sigma(I_o)$, R_{int} = 0.110, 3166 data, 206 parameters, 198 restraints, GooF = 1.33, 0.60 > Δρ > -0.53 e$Å^{-3}$, $R[F^2 > 2\sigma(F^2)]$ = 0.136, $wR(F^2)$ = 0.399, CCDC 1530418.

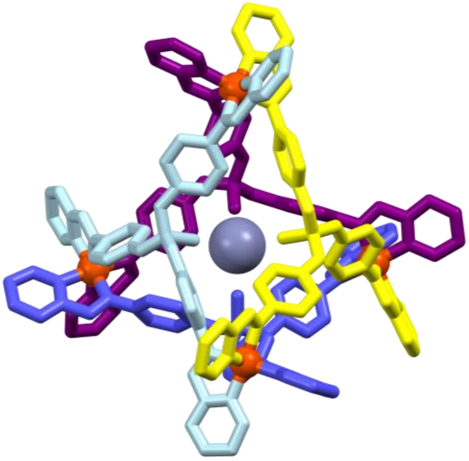

Figure S3. The molecular structure of **$[Zn]^{2+}$@$[Fe_4L_4][BF_4]_{10}$** (anions and hydrogen atoms omitted for clarity; thermal ellipsoids at 50% probability).

SCXRD Tables

Table S1. The single-crystal X-ray diffraction details of all new crystal structures reported herein.

Complex	PL1	H_2O@[Fe_4L_4][BF_4]$_8$	[BF_4]$^-$@[Fe_4L_4][BF_4]$_7$	[Cu]$^+$@[Fe_4L_4][BF_4]$_9$	[Zn]$^{2+}$@[Fe_4L_4][BF_4]$_{10}$
Formula	$C_{18}H_{18}N_3O_4P$	$C_{144}H_{110}B_8F_{32}Fe_4N_{24}O_{17}P_4$	$C_{172}H_{147}B_8F_{32}Fe_4N_{32}O_{16}P_4$	$C_{144}H_{109}B_{8.50}Cu_{0.50}F_{34}Fe_4N_{24}O_{16.50}P_4$	$C_{144}H_{108}B_{10}F_{40}Fe_4N_{24}O_{16}P_4Zn$
Formula Weight (gmol^{-1})	371.32	3490.31	3959.97	3556.48	3711.29
Colour & Habit	Yellow plate	Purple block	Purple block	Purple block	Purple block
Crystal Dimensions (mm)	0.03 x 0.18 x 0.27	0.10 x 0.10 x 0.10	0.11 x 0.32 x 0.57	0.09 x 0.17 x 0.23	0.08 x 0.14 x 0.17
Crystal System	Triclinic	Cubic	Orthorhombic	Cubic	Cubic
Space Group	*P*-1 (No. 2)	*I*23	*Iba*2	*I*23	*I*23
a (Å)	9.307(1)	21.010(2)	56.1140(8)	20.9262(10)	21.0609(12)
b (Å)	9.8081(8)	21.010(2)	28.1044(4)	20.9262(10)	21.0609(12)
c (Å)	10.3920(7)	21.010(2)	24.1794(3)	20.9262(10)	21.0609(12)
α (°)	67.018(7)	90	90	90	90
β (°)	81.591(8)	90	90	90	90
γ (°)	83.423(8)	90	90	90	90
V (Å)	862.21(14)	9275(3)	38132.1(9)	9163.7(13)	9341.8(16)
Z	2	2	8	2	2
ρ_{calc} (gcm^3)	1.430	1.250	1.380	1.289	1.319
F(000)	388	3540	16184	3600	3744
μ (mm^{-1})	1.68	0.43	3.56	3.70	3.77
Temperature (K)	120.0(1)	100(2)	120.0(1)	120.0(1)	120.0(1)
ϑ_{max} [°]	76.5	26.3	77.1	43.3	75.3
Total Reflections	6019	33249	181371	12837	32886
Independent Reflections	3490	3141	34718	1105	3166
Reflections ($I_o > 2\sigma[I_o]$)	2837	1816	29090	921	1717
R_{int}	0.037	0.130	0.104	0.092	0.110
Parameters	258	259	2491	194	0.110
Restraints	1	220	519	226	198
GooF (F^2)	1.05	1.33	1.02	2.19	1.33
R_1 ($I_o > 2\sigma[I_o]$)	0.0498	0.1179	0.0799	0.1699	0.1363
R_1 (all reflections)	0.0612	0.1693	0.0959	0.1810	0.1806
wR_2 ($I_o > 2\sigma[I_o]$)	0.1266	0.3102	0.2046	0.4192	0.3494
wR_2 (all reflections)	0.1392	0.3673	0.2202	0.4454	0.3985
Largest Peak (eÅ^{-3})	0.27	0.46	1.77	0.65	0.60
Largest Hole (eÅ^{-3})	-0.40	-0.50	-0.90	-0.59	-0.53
CCDC Number	1530372	1530416	1530371	1530417	1530418

High Resolution Mass Spectroscopy

***Tris*(4-aminophenyl)phosphate (PL1)**

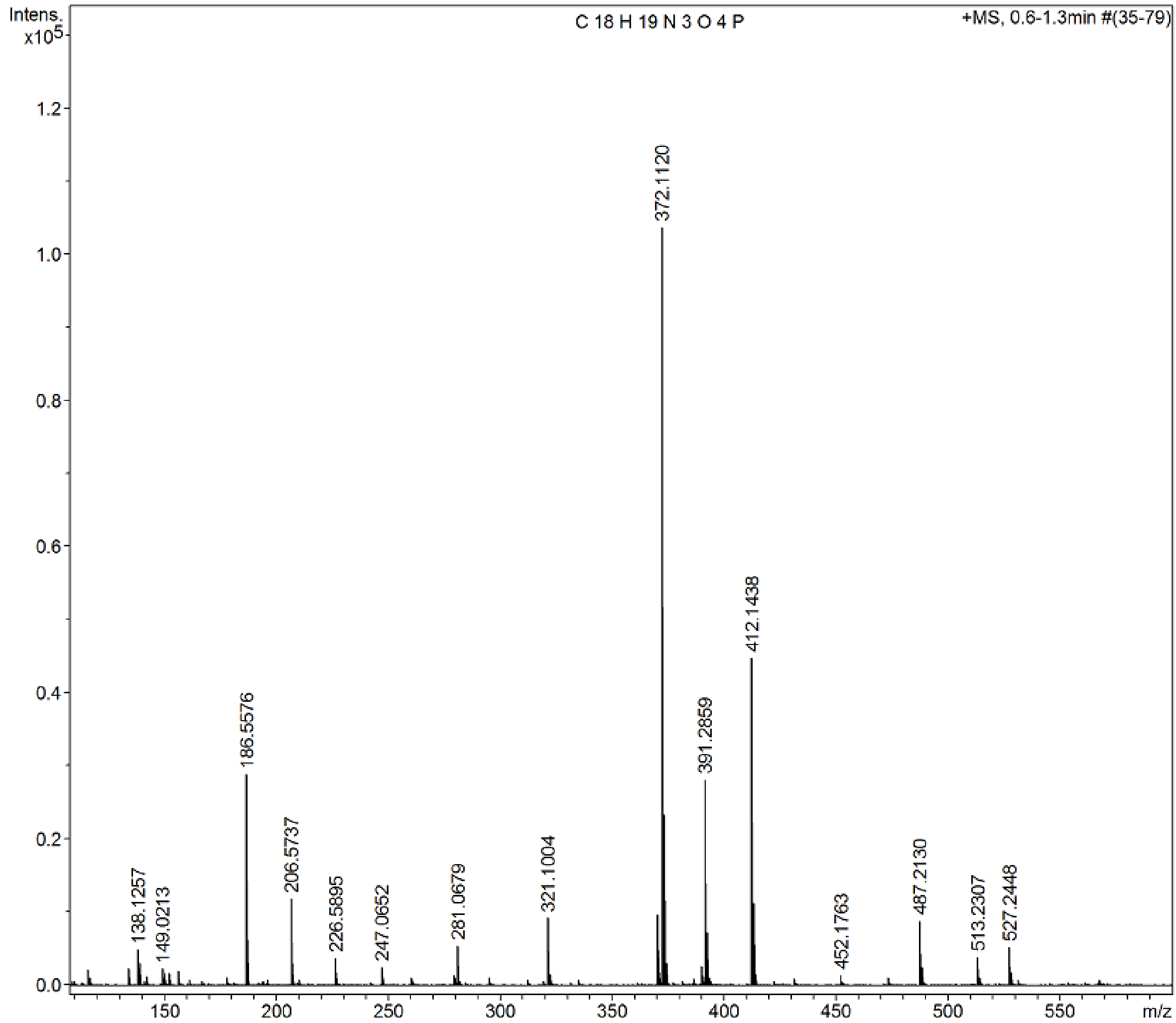

Figure S4. The experimental mass spectrum of $C_{18}H_{18}N_3O_4P$ (in MeOH).

H_2O@[Fe_4L_4][BF_4]$_8$

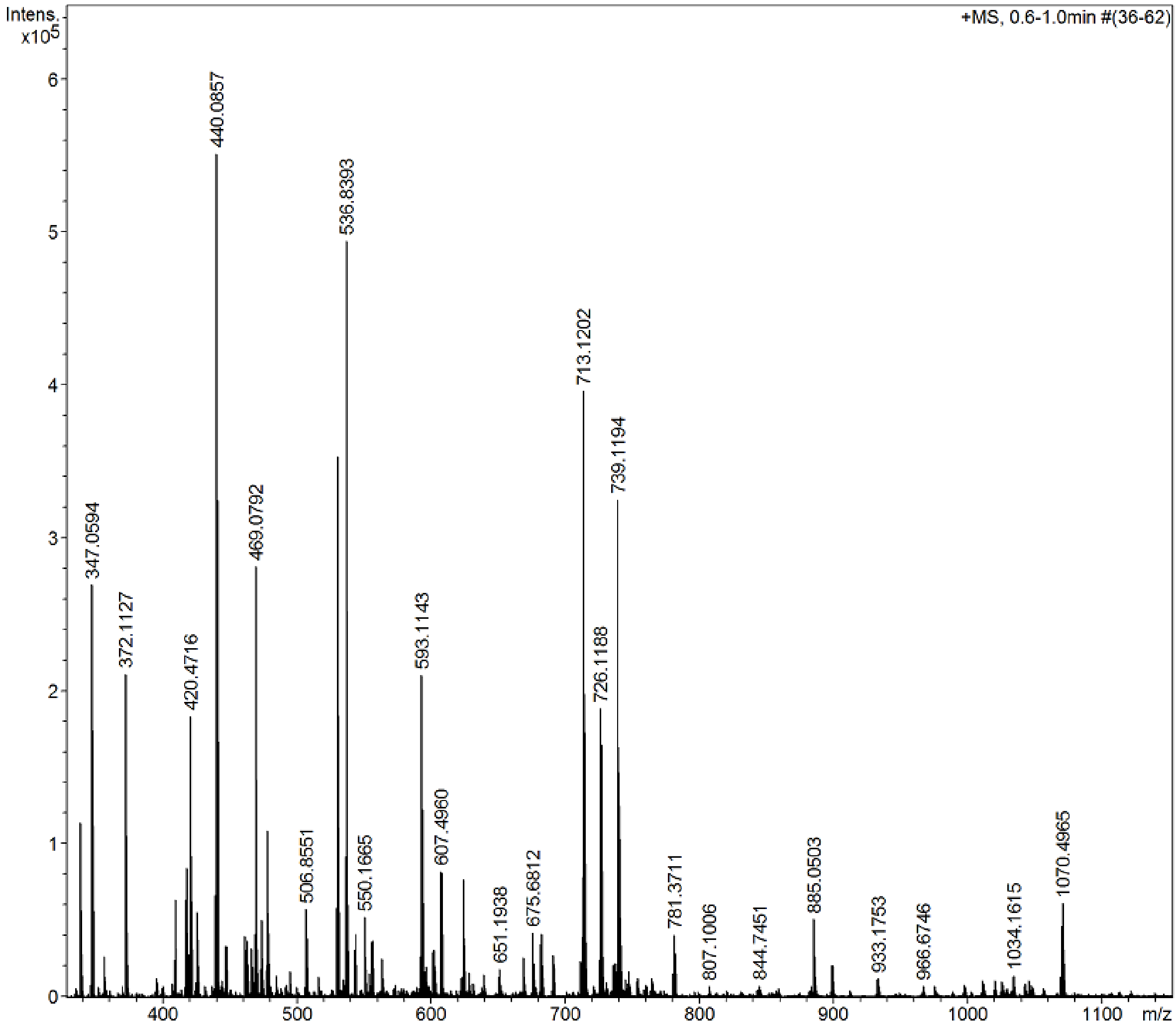

Figure S5. The experimental mass spectrum of **H_2O@[Fe_4L_4][BF_4]$_8$** (in MeCN).

H_2O@[Fe_4L_4][BF_4]$_8$

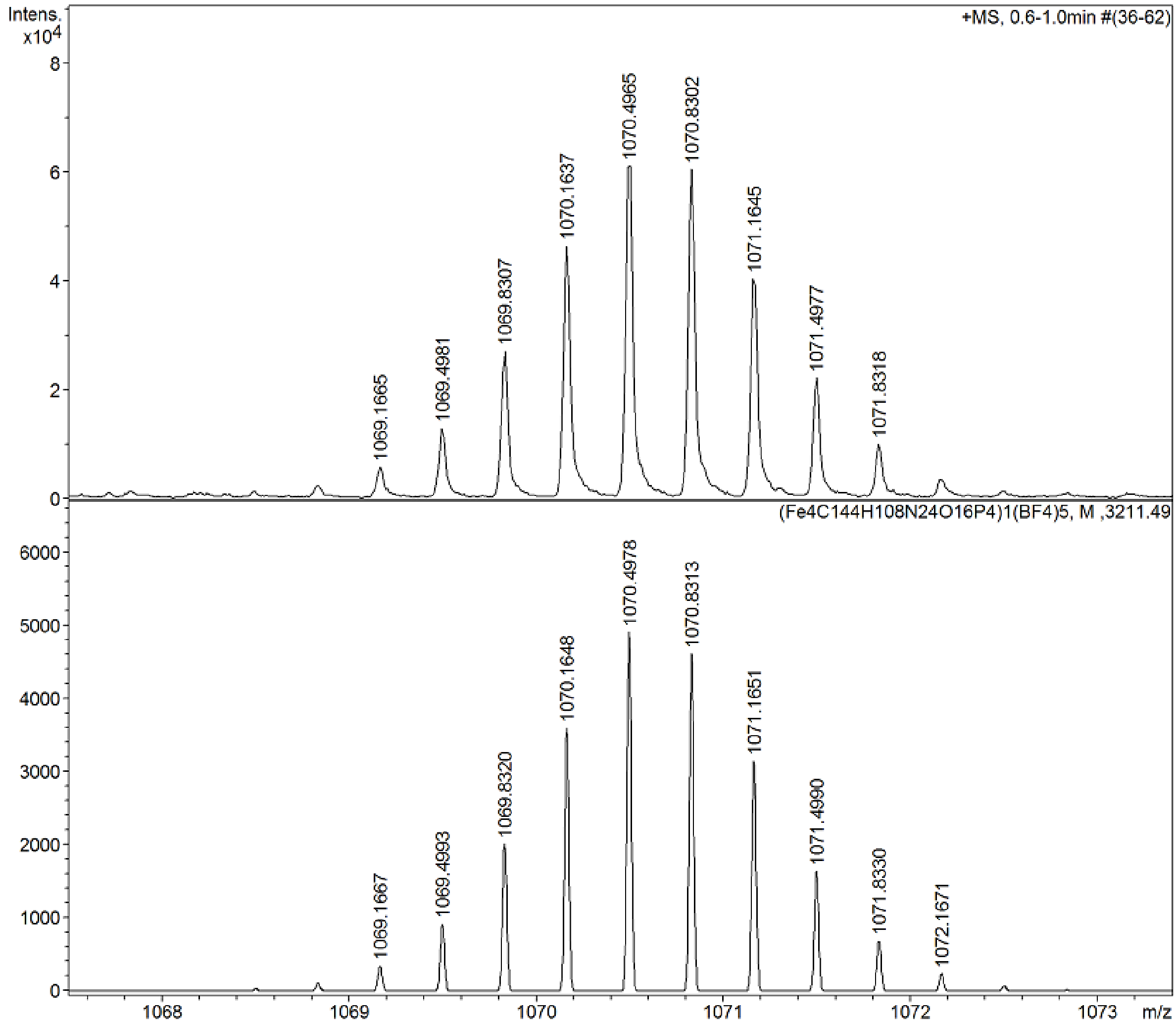

Figure S6. The experimental (above) and simulated (below) mass spectrum for [Fe_4L_4 + $(BF_4)_5$]$^{3+}$.

$H_2O@[Fe_4L_4][BF_4]_8$

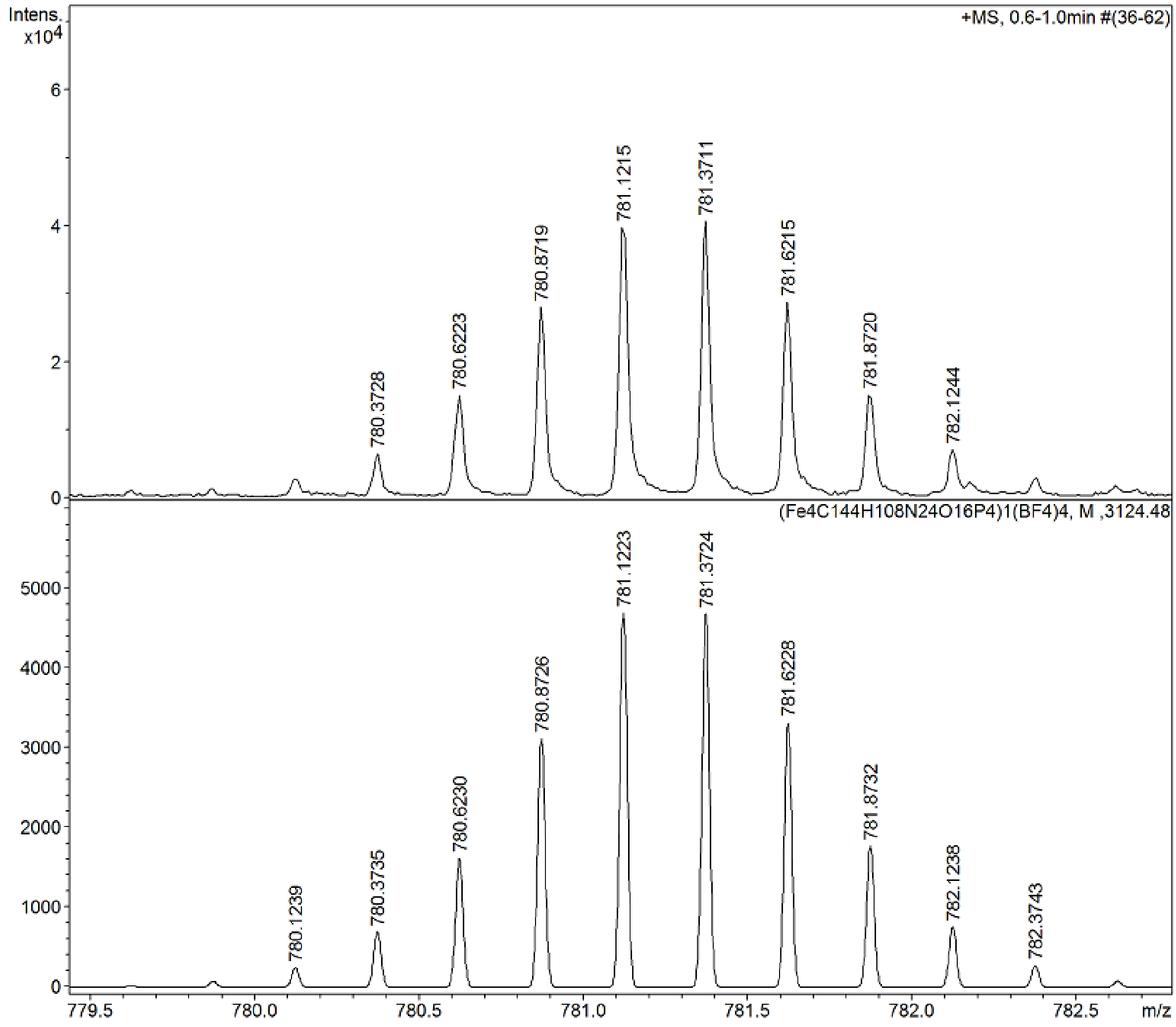

Figure S7. The experimental (above) and simulated (below) mass spectrum for $[Fe_4L_4 + (BF_4)_4]^{4+}$.

H_2O@[Fe_4L_4][BF_4]$_8$

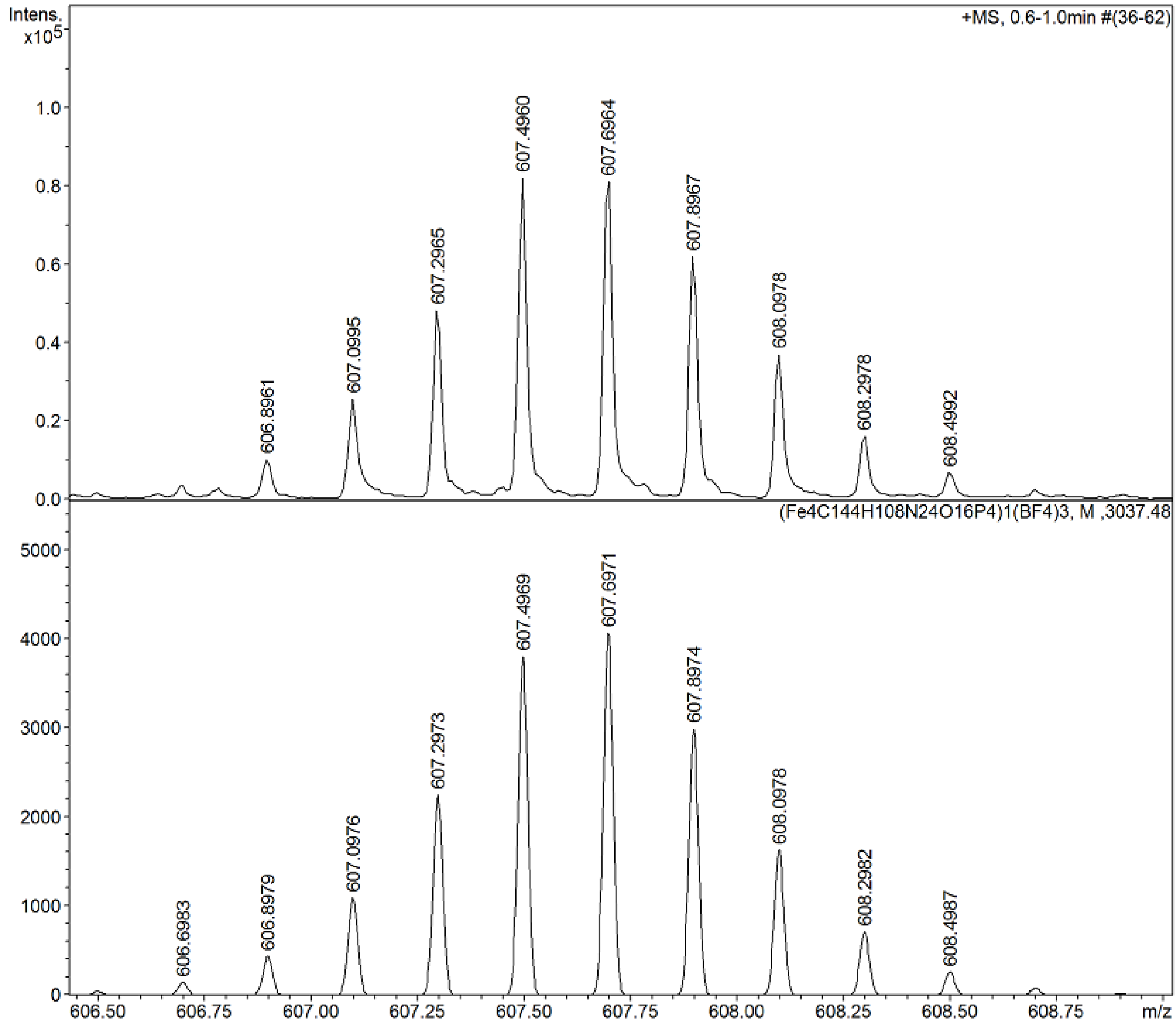

Figure S8. The experimental (above) and simulated (below) mass spectrum for [Fe_4L_4 + $(BF_4)_3$]$^{5+}$.

H_2O@[Fe_4L_4][BF_4]$_8$

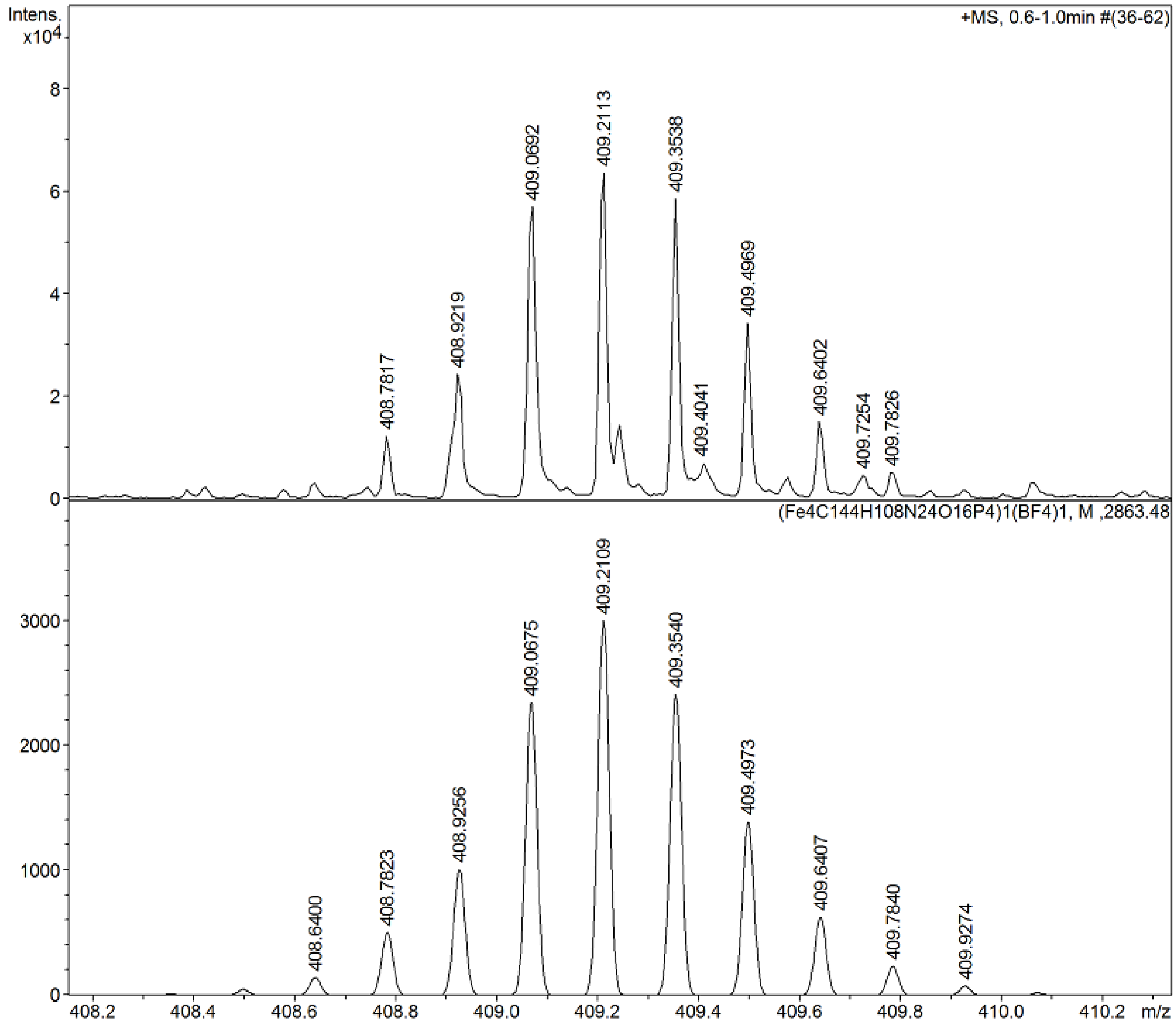

Figure S9. The experimental (above) and simulated (below) mass spectrum for [Fe_4L_4 + BF_4]$^{7+}$.

$[BF_4]^-$@$[Fe_4L_4][BF_4]_7$

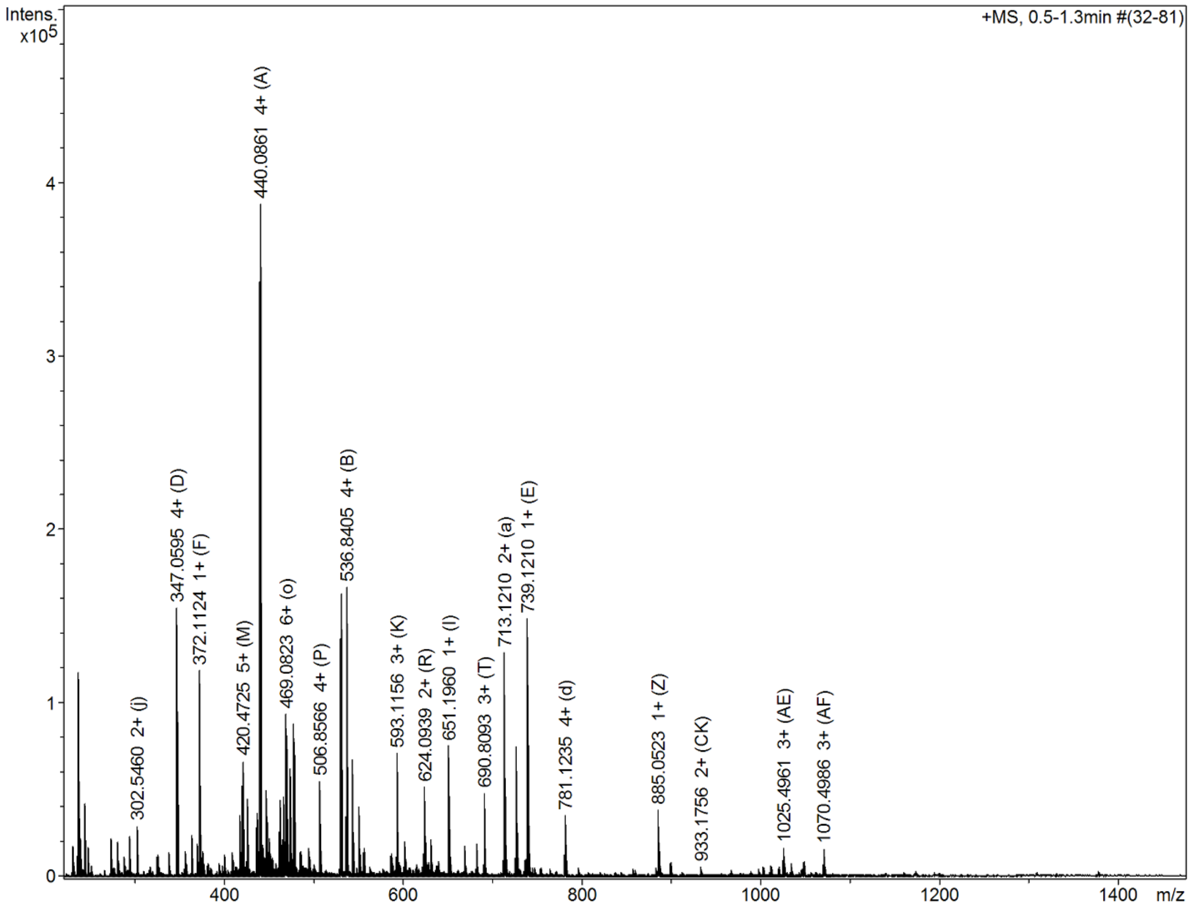

Figure S10. The experimental mass spectrum of **H_2O@$[Fe_4L_4][BF_4]_8$** (in MeCN)

$[BF_4]^-$@$[Fe_4L_4][BF_4]_7$

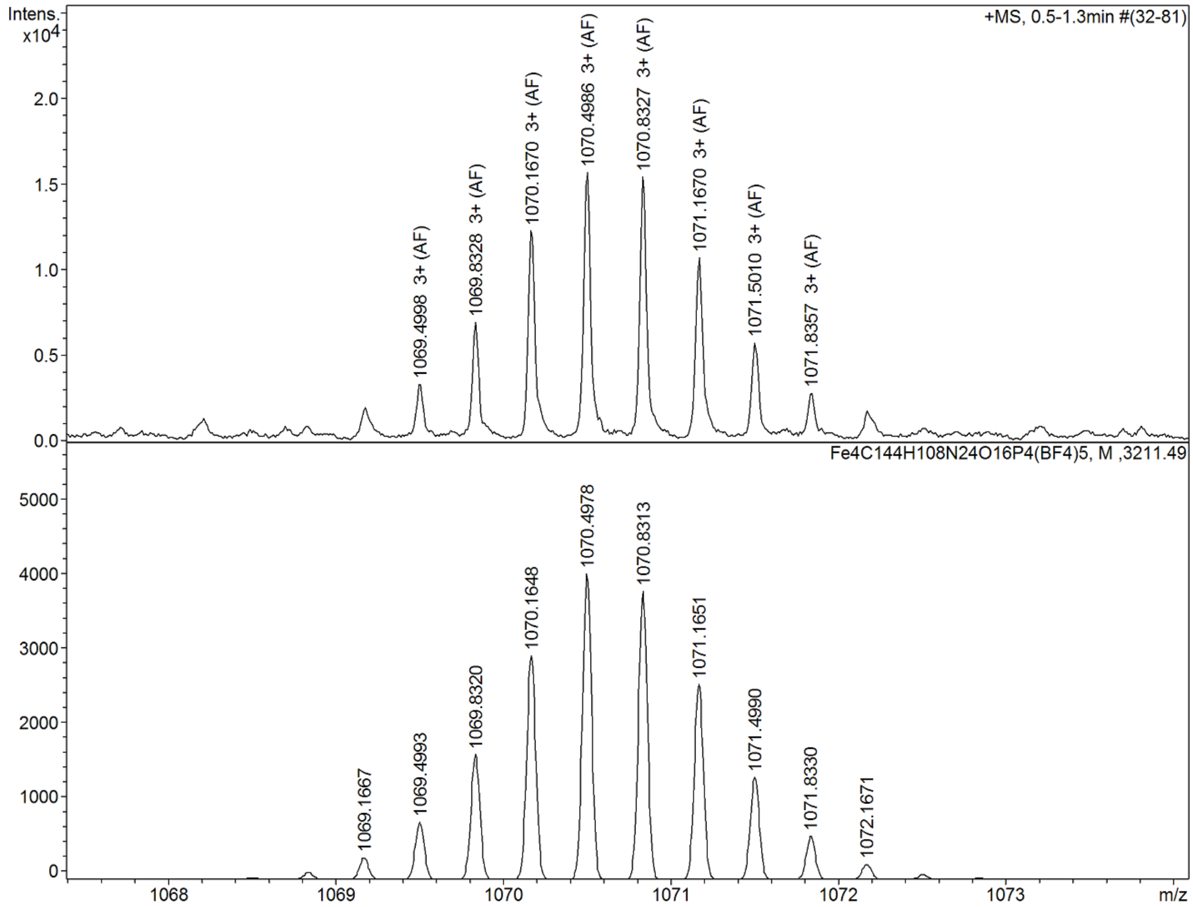

Figure S11. The experimental (above) and simulated (below) mass spectrum for $[Fe_4L_4 + (BF_4)_5]^{3+}$.

$[BF_4]^-@[Fe_4L_4][BF_4]_7$

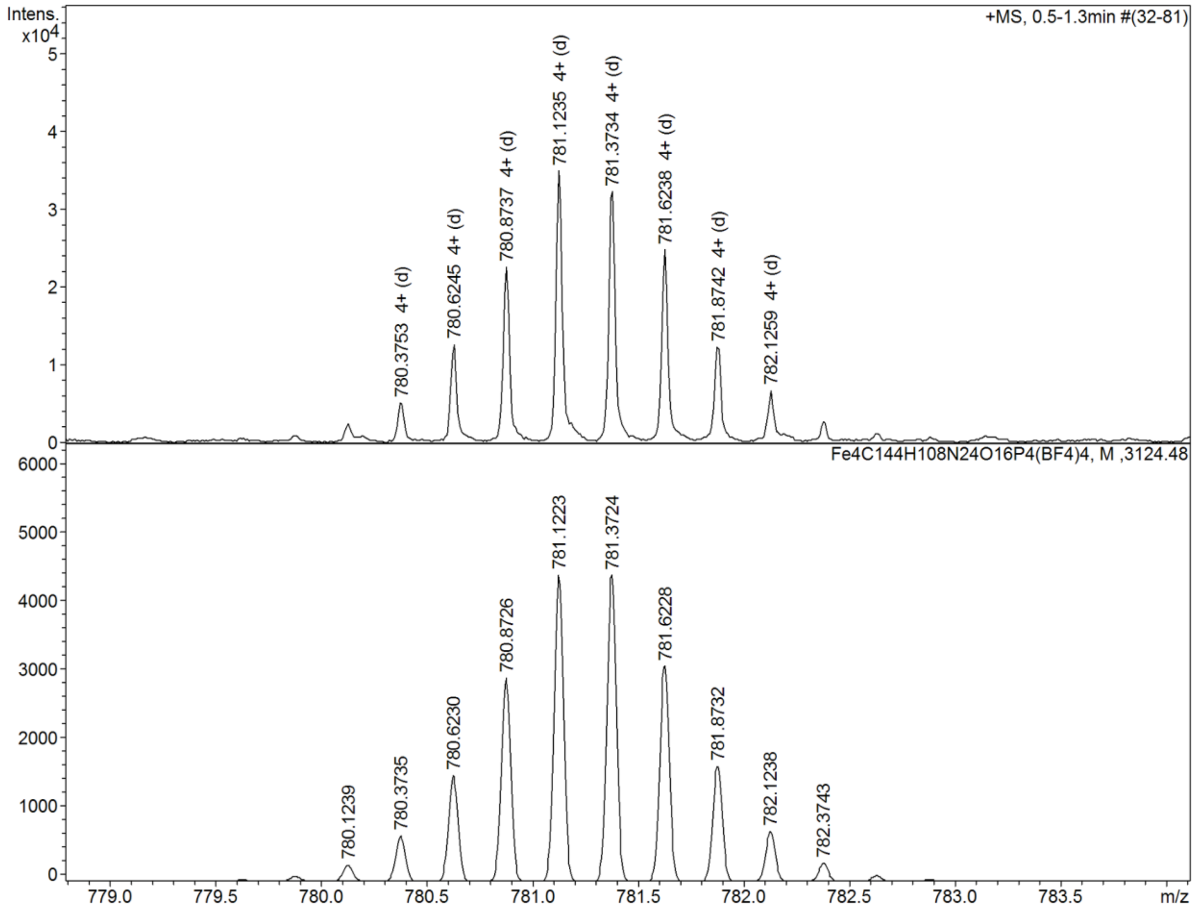

Figure S12. The experimental (above) and simulated (below) mass spectrum for $[Fe_4L_4 + (BF_4)_4]^{4+}$.

[Zn^{2+}]@[Fe$_4$L$_4$][BF$_4$]$_{10}$

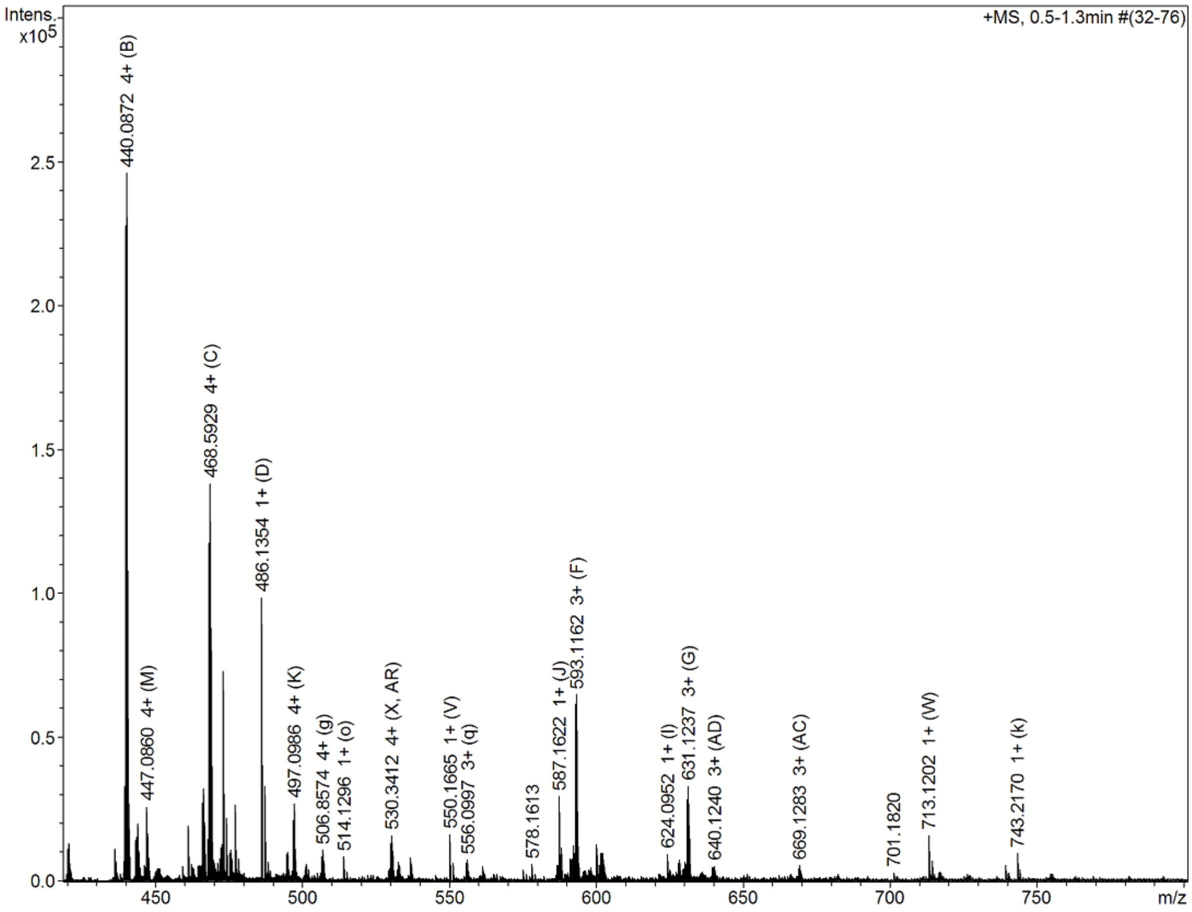

Figure S13. The experimental mass spectrum of **[Zn^{2+}]@[Fe$_4$L$_4$][BF$_4$]$_{10}$** (in MeCN).

[Zn^{2+}]@[Fe_4L_4][BF_4]$_{10}$

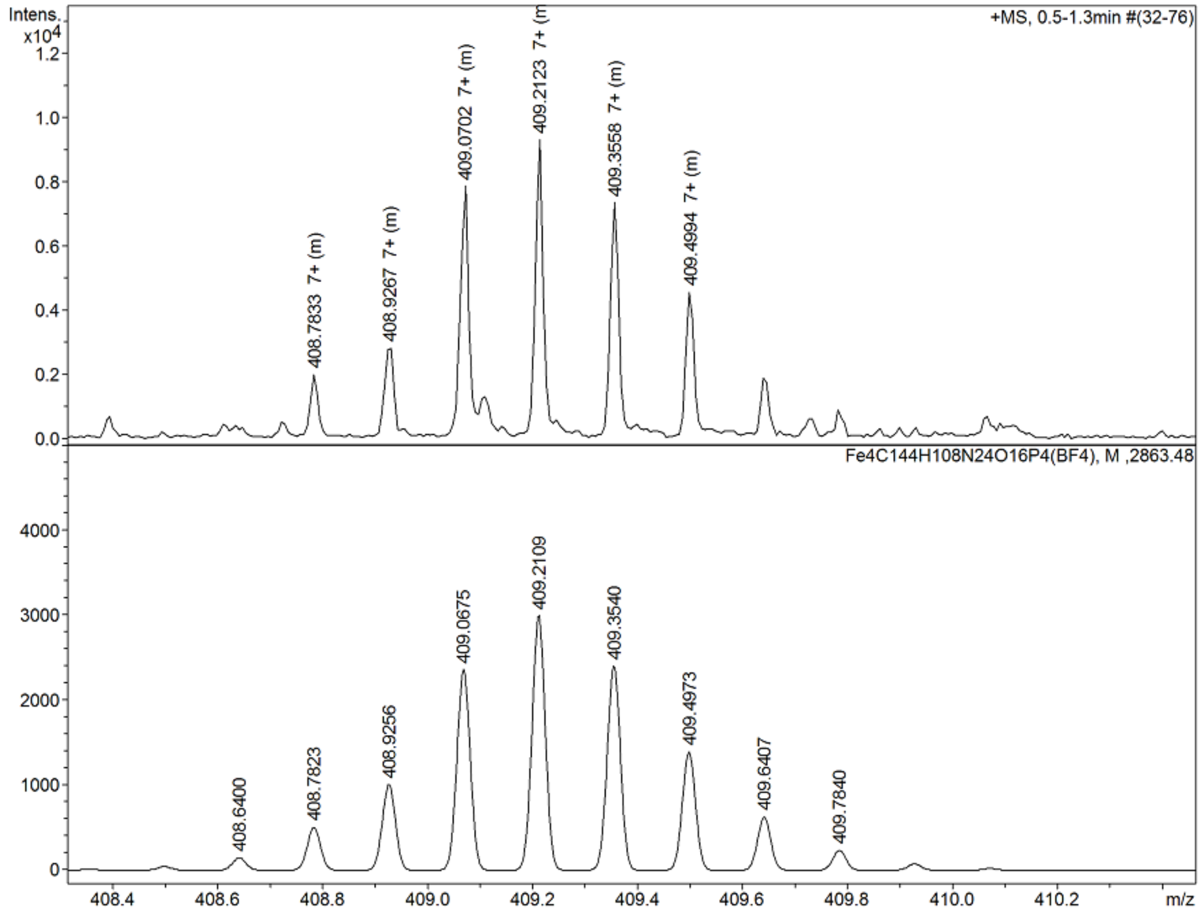

Figure S14. The experimental (above) and simulated (below) mass spectrum for [Fe_4L_4 + BF_4]$^{7+}$.

NMR Spectroscopy

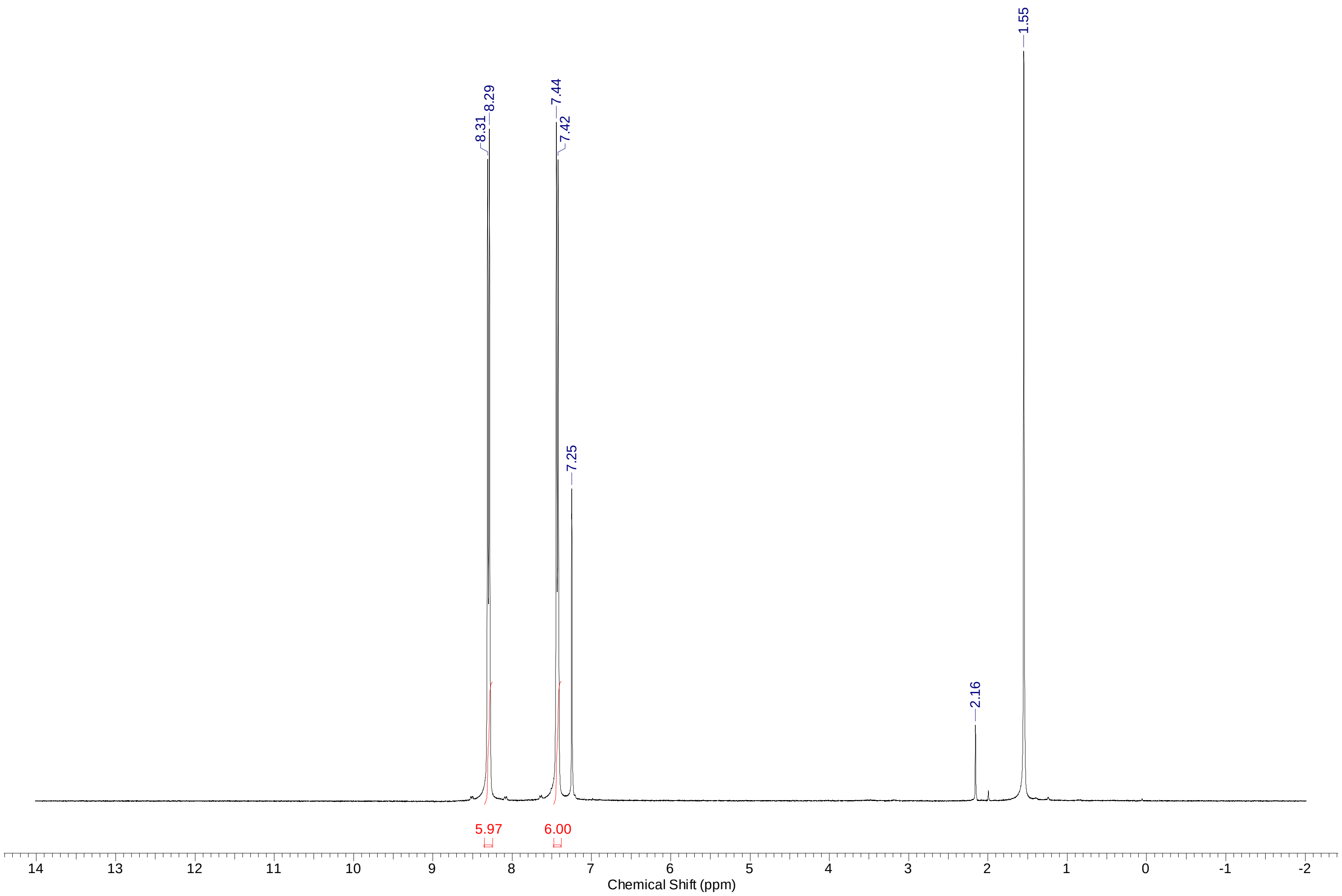

Figure S15. The ¹H NMR spectrum for *tris*(4-nitrophenyl)phosphate (in $CDCl_3$).

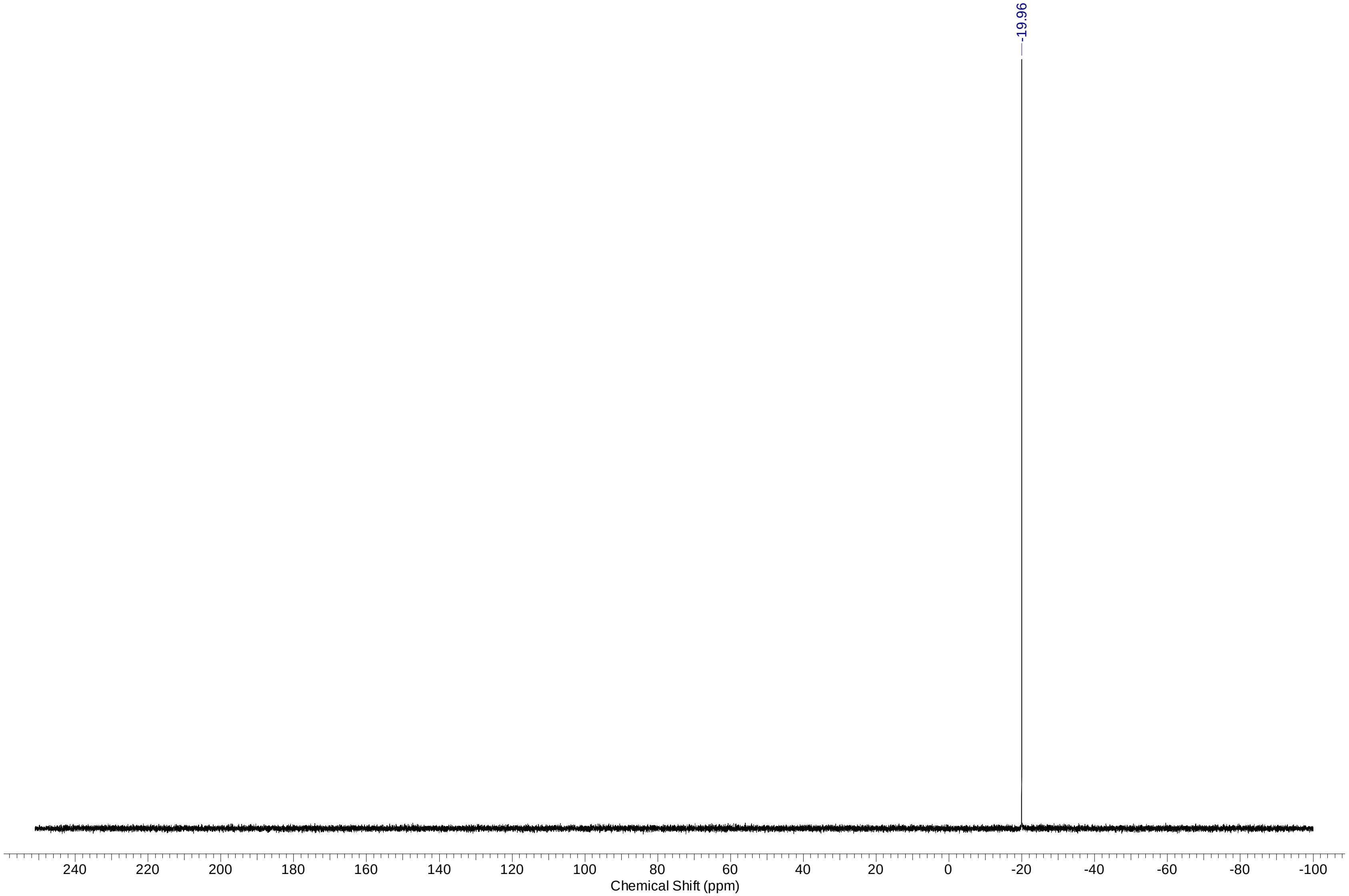

Figure S16. The ^{31}P NMR spectrum for *tris*(4-nitrophenyl)phosphate (in $CDCl_3$).

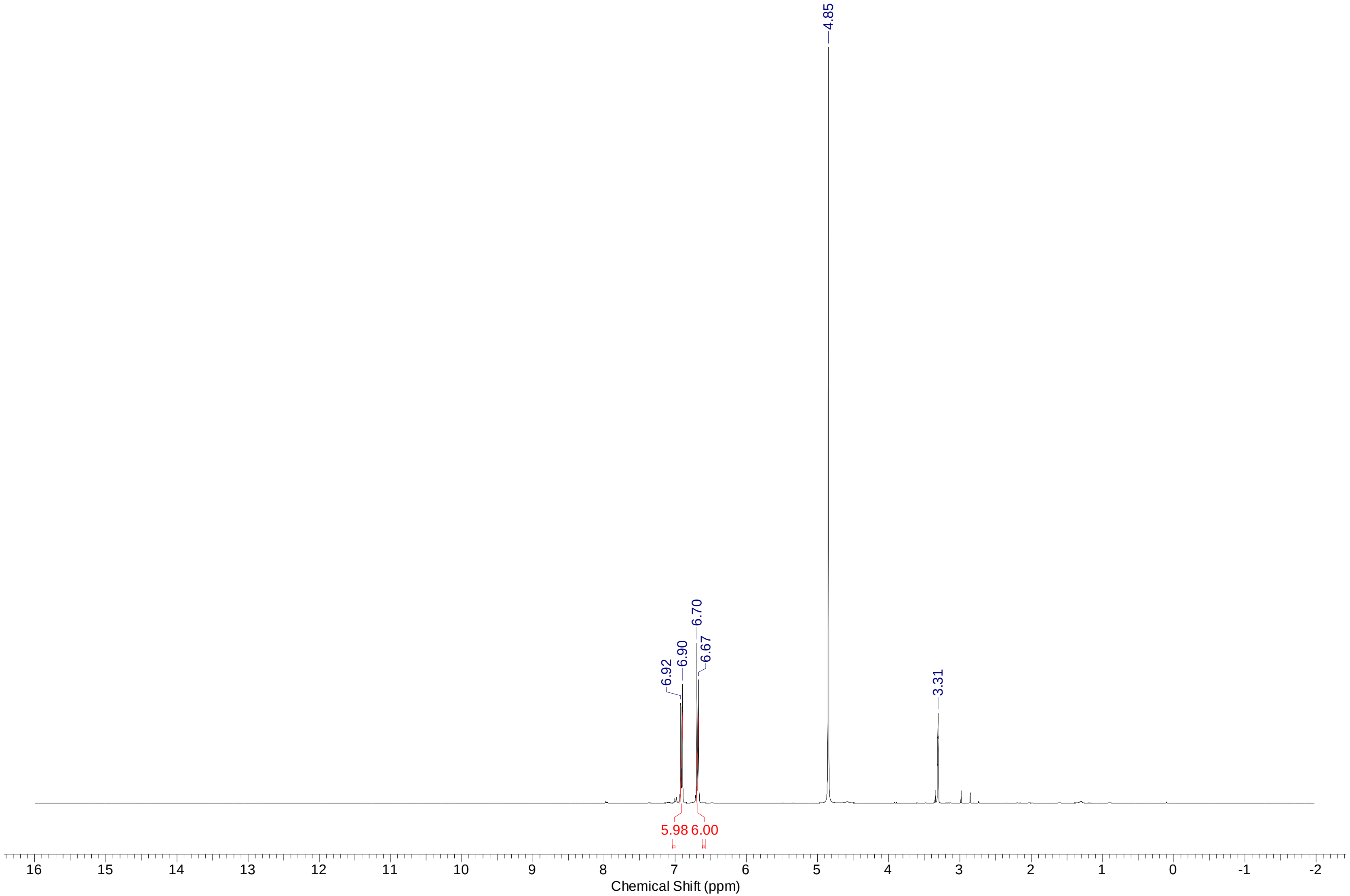

Figure S17. The ^{1}H NMR spectrum for *tris*(4-aminophenyl)phosphate (**PL1**; in CD_3OD).

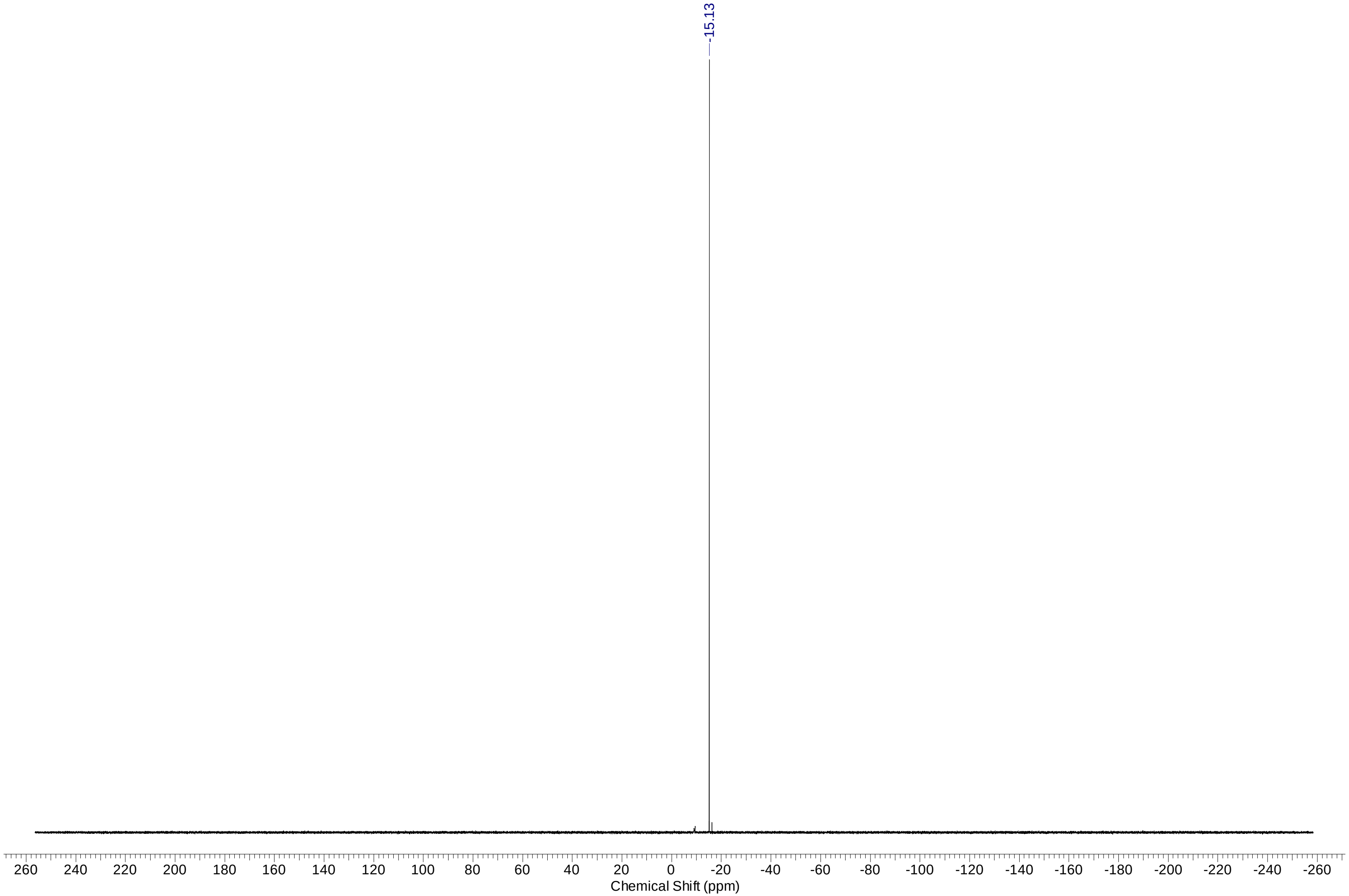

Figure S18. The ^{31}P NMR spectrum for *tris*(4-aminophenyl)phosphate (**PL1**; in CD_3OD).

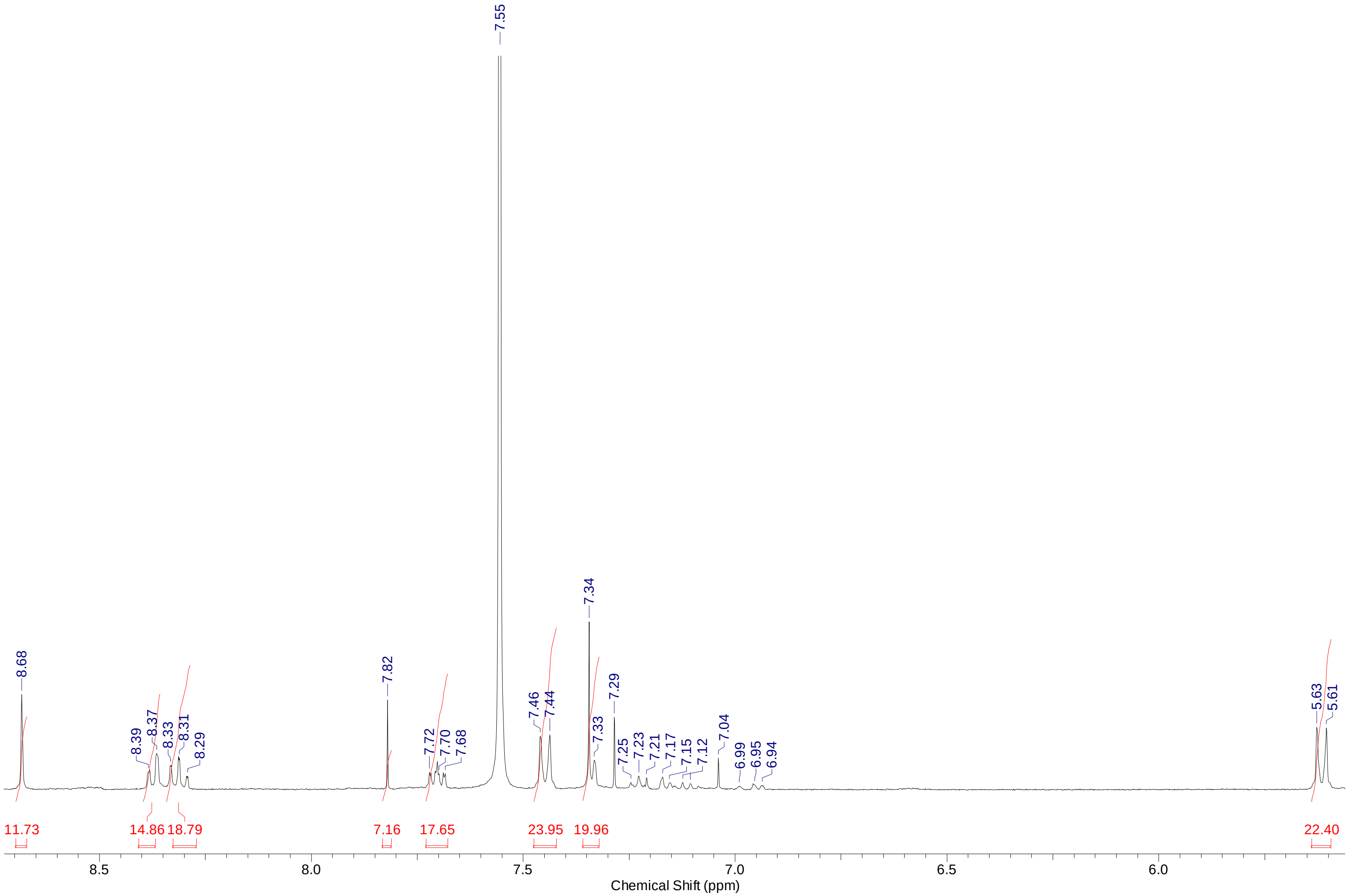

Figure S19. The ^{1}H NMR spectrum for $[BF_4]^-$@$[Fe_4L_4][BF_4]_7$ (in CD_3CN).

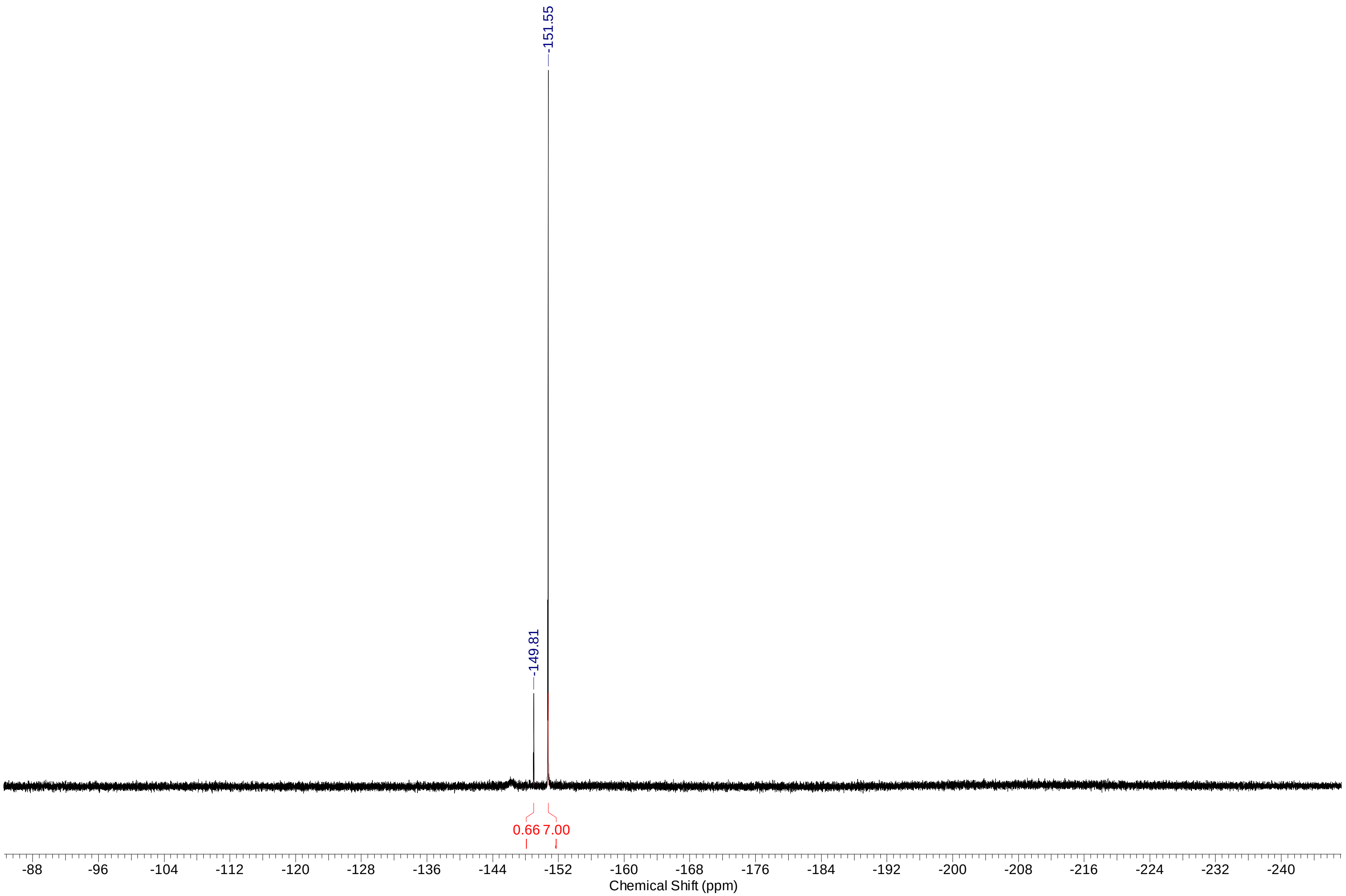

Figure S20. The ^{19}F NMR spectrum for $[BF_4]^-@[Fe_4L_4][BF_4]_7$ (in CD_3CN).

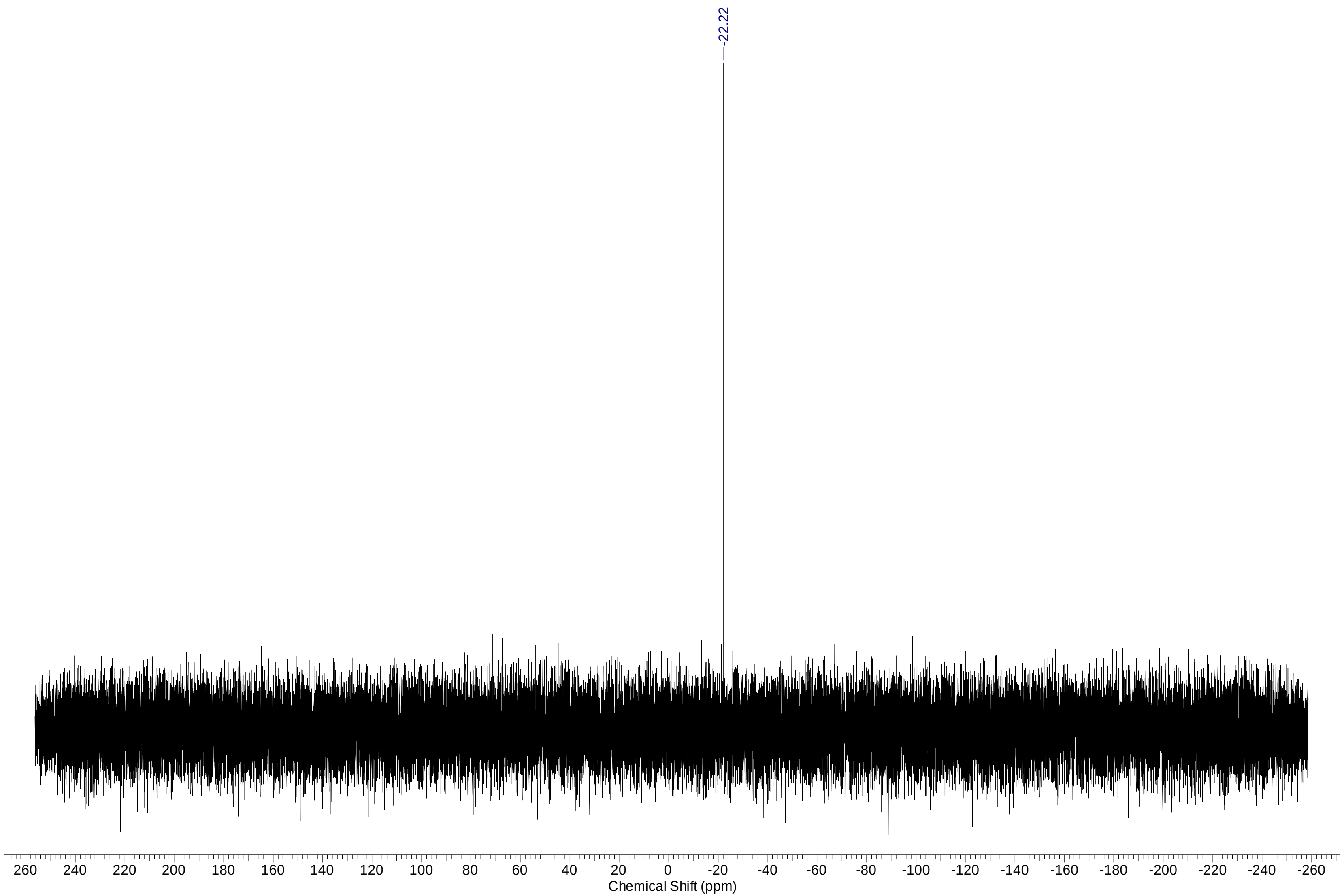

Figure S21. The ^{31}P NMR spectrum for $[BF_4]^-@[Fe_4L_4][BF_4]_7$ (in CD_3CN).

References

1 W. Kabsch, *J. Appl. Crystallogr.*, 1993, **26**, 795–800.

2 T. M. McPhillips, S. E. McPhillips, H.-J. Chiu, A. E. Cohen, A. M. Deacon, P. J. Ellis, E. Garman, A. Gonzalez, N. K. Sauter, R. P. Phizackerley, S. M. Soltis and P. Kuhn, *J. Synchrotron Radiat.*, 2002, **9**, 401–406.

3 G. M. Sheldrick, *Acta Crystallogr. Sect. A Found. Adv.*, 2015, **71**, 3–8.

4 G. M. Sheldrick, *Acta Crystallogr. Sect. C, Struct. Chem.*, 2015, **71**, 3–8.

5 O. V. Dolomanov, L. J. Bourhis, R. J. Gildea, J. A. K. Howard and H. Puschmann, *J. Appl. Crystallogr.*, 2009, **42**, 339–341.

6 A. J. Kirby, M. Medeiros, P. S. M. Oliveira, E. S. Orth, T. A. S. Brandao, E. H. Wanderlind, A. Amer, N. H. Williams and F. Nome, *Chem. - A Eur. J.*, 2011, **17**, 14996–15004.
